# PA2G4 Functions as a Cofactor for MYC Family Oncoproteins in MYC-Driven Malignancies

**DOI:** 10.3390/cells14181422

**Published:** 2025-09-11

**Authors:** Sukriti Krishan, Jessica Koach, Taylor Lim, Kenny Yeo, Faith Cheong, Jie-Si Luo, Hassina Massudi, Xiaomian Gao, Sopheakwealthy Heangsarath, Andrew J. Kueh, Marco J. Herold, Belamy B. Cheung, Glenn M. Marshall

**Affiliations:** 1 Children’s Cancer Institute Australia for Medical Research, Lowy Cancer Research Centre, University of New South Wales Sydney (UNSW), Sydney, NSW 2052, Australia; skrishan@ccia.org.au (S.K.); jessica.koach@arodia.com (J.K.); xinyilim.06@gmail.com (T.L.); kyeo@ccia.org.au (K.Y.); luojs5@mail.sysu.edu.cn (J.-S.L.); swealthysheang@gmail.com (S.H.); 2 School of Clinical Medicine, UNSW Medicine & Health, University of New South Wales Sydney (UNSW), Sydney, NSW 2052, Australia; 3 Olivia Newton-John Cancer Research Institute, Heidelberg, Melbourne, VIC 3086, Australia; andrew.kueh@onjcri.org.au (A.J.K.); marco.herold@onjcri.org.au (M.J.H.); 4 School of Cancer Medicine, La Trobe University, Heidelberg, Melbourne, VIC 3000, Australia; 5 The Walter and Eliza Hall Institute of Medical Research, Parkville, Melbourne, VIC 3050, Australia; 6 Department of Medical Biology, University of Melbourne, Parkville, Melbourne, VIC 3010, Australia; 7 Kids Cancer Centre, Sydney Children’s Hospital, Randwick, Sydney, NSW 2031, Australia

**Keywords:** MYC, MYCN, PA2G4, neuroblastoma, Burkitt’s lymphoma, inhibitors, WS6

## Abstract

MYCN and c-MYC are critical driver oncogenes in several childhood cancers, including neuroblastoma. Currently, the clinical development of MYC inhibitors has been hindered by the intrinsically disordered structure of MYC proteins, which lack well-defined ligand-binding pockets. Proliferation-associated protein 2G4 (PA2G4) directly binds to and stabilizes MYCN protein, leading to markedly increased MYCN levels in neuroblastoma cells. Here, we demonstrate that PA2G4 is essential for MYCN-driven tumor growth in neuroblastoma in vivo. Moreover, PA2G4 elevates c-MYC protein levels in neuroblastoma cells by inhibiting its ubiquitin-mediated degradation. In turn, c-MYC upregulates the transcription and protein expression of PA2G4, creating an oncogenic feed-forward expression loop. A small molecule PA2G4 inhibitor, WS6, directly disrupts the PA2G4-c-MYC protein–protein interaction, resulting in decreased levels of both PA2G4 and c-MYC. WS6 exhibited selective cytotoxicity in c-MYC-overexpressing cell lines. Together, these findings identify PA2G4 as a shared cofactor for both the c-MYC and MYCN oncoproteins and highlight its interaction with MYC family oncoproteins as a promising therapeutic vulnerability in MYC-driven cancers.

## 1. Introduction

The *MYC* oncogene is deregulated in more than 50% of human cancers, including both adult and pediatric malignancies [1,2]. The *MYC* family of oncogenes (*c-MYC*, *MYCN*, and *L-MYC*) encode transcription factors, expressed ubiquitously across cells [3]. These transient “super-transcription factors” regulate over 15% of the human genome, controlling crucial cellular processes such as metabolism [4], proliferation [5], differentiation [6], and apoptosis [7].

Despite their critical role in oncogenesis, direct therapeutic targeting of MYC proteins has proven difficult, as it is an intrinsically disordered protein without a specific active site and with no hydrophobic pockets [2]. As a transcription factor, MYC is located in the nucleus, making it inaccessible to monoclonal antibody therapies [1]. Consequently, MYC has been labeled “undruggable,” and research has primarily focused on indirect approaches for targeting its function [1].

In recent years, extensive efforts have been made to inhibit MYC at various stages of its biogenesis and activity [8,9,10]. These include targeting *MYC* gene transcription, MYC mRNA translation, MYC/Max interaction [11], MYC protein stability [8], and MYC downstream pathways [12]. We previously identified a novel MYCN binding protein known as proliferation-associated protein 2G4 (PA2G4), which stabilizes MYCN and prevents its degradation [12]. One mechanism by which PA2G4 can cause MYC stabilization is by sequestering Fbxw7, a component of the ubiquitin–proteasome pathway [13], in the cytoplasm. In neuroblastoma cells, PA2G4 and MYCN act together in a forward feedback loop, driving continued tumorigenesis [12]. High PA2G4 expression independently predicts poor patient survival. Targeting PA2G4/MYCN binding by WS6, a small molecule inhibitor, leads to reduced levels of both proteins and suppresses neuroblastoma tumorigenicity in vitro and in vivo [12,14].

While the PA2G4-MYCN interaction in neuroblastoma has been well characterized [12], little is known about its potential role in regulating c-MYC. Notably, in high-risk neuroblastoma cases lacking *MYCN* amplification, c-MYC overexpression can serve as a functional driver, conferring similarly poor clinical outcomes [15]. *MYCN* and *c-MYC* share significant structural and functional homology [16], with both genes composed of three exons interrupted by two introns and showing 84% amino acid identity in common regions. In mouse models, *MYCN* can compensate for the normal functions of *c-MYC*, supporting survival, development, and reproduction in the absence of c-MYC [16]. This functional redundancy suggests a conserved oncogenic role for PA2G4 across MYC family members and raises the possibility that PA2G4 may also contribute to tumorigenesis through stabilization of c-MYC in c-MYC-driven malignancies.

In this study, we reveal PA2G4 as a critical cofactor for both MYCN and c-MYC in neuroblastoma. We demonstrate that PA2G4 promotes MYCN-driven tumor growth in vivo and stabilizes c-MYC protein by preventing its proteasomal degradation. Furthermore, we identify a feed-forward loop wherein c-MYC transcriptionally upregulates PA2G4 expression. Targeting this oncogenic axis with the small-molecule inhibitor WS6 disrupts the PA2G4–c-MYC interaction, reduces oncoprotein levels, and selectively impairs the viability of c-MYC-overexpressing cells. These findings establish PA2G4 as a shared vulnerability in MYC-driven neuroblastoma and a promising therapeutic target.

## 2. Materials and Methods

### 2.1. Cell Lines

Neuroblastoma cell lines (SH-SY5Y, SK-N-AS) and two normal lung fibroblast cell lines (MRC-5 and WI-38) were obtained from the American Type Culture Collection (ATCC). SH-SY5Y and SK-N-AS cells were maintained in Dulbecco’s modified Eagle’s medium (DMEM) (Invitrogen Life Technologies, Carlsbad, CA, USA) with 10% fetal Bovine serum (FBS). MRC-5 and WI-38 were cultured in Alpha-MEM media (Invitrogen, Life Technologies) with 10% FBS. Human B-Acute Lymphoblastic Leukemia (B-ALL) cell lines; KOPN-8, SEM-K2, and human Burkitt’s Lymphoma (BL) cell lines; and Raji and P493-6 were maintained in Roswell Park Memorial Institute (RPMI) 1640 medium (Life Technologies) supplemented with 10% FBS. Murine B-cell ALL cell lines WEHI-47 and WEHI-116, derived from the Eµ-Myc mouse model (Walter and Eliza Hall Institute of Medical Research, Parkville, Australia), were maintained in DMEM supplemented with 10% FBS, 100 μM L-aspafbragine, and 55 μM β-metacaptoethanol. Medulloblastoma cell line UW-288 was obtained from Dr Nick Gottardo (Telethon Kids Institute, Nedlands, Australia) and maintained in DMEM supplemented with 10% FBS. All cell lines used were authenticated by Cell Bank Australia (Westmead, Australia), free from mycoplasma, and cultured at 37 °C and 5% CO_2_ in a humidifier incubator.

The P493-6 cell line was established from the stable transfection of EBNA2-conditional EREB2-5 cells (B-cells), with a c-MYC expression construct (pmyc-tet), and was provided by Stefania Purgato, Dipartimento di Farmacia e Biotecnologie (FABIT, Bologna, Italy). To suppress the expression of c-MYC in P493-6 cells, 1 µg/mL doxycycline (Life Technologies) was added to cells growing in RPMI medium. P493-6 cells treated with doxycycline to repress c-MYC are referred to as P493-6 +Dox, and untreated cells are referred to as P493-6 -Dox.

### 2.2. Cell Viability

Cell viability was assessed using resazurin. The reagent was prepared in phosphate-buffered saline (PBS) and contained 75 mg resazurin, 12.5 mg methylene blue, 164.5 mg potassium hexacyanoferrate (III), and 211 mg potassium hexacyanoferrate (II) trihydrate in 500 mL of PBS. After 6 h incubation with resazurin, the change in fluorescence was measured by Victor 3 multilabel Plate Reader (Perkin Elmer, Shelton, CT, USA) at an excitation wavelength of 560 nm and an emission wavelength of 590 nm.

### 2.3. Western Blot

Protein was extracted from cell pellets using RIPA buffer (Sigma-Aldrich, Burlington, MA, USA) with 10% protease inhibitor (Sigma-Aldrich). Quantification of proteins was conducted using the Pierce BCA protein assay kit (Thermo Scientific, Waltham, MA, USA) according to the manufacturer’s instructions. A total of 30–50 μg protein samples were loaded onto Criterion TGX 10% precast gel (Bio-Rad, Gladesville, Australia) and transferred to a nitrocellulose membrane (Bio-Rad). The membrane was blocked in 5% skim milk in Tris-buffered saline with Tween-20 (20 mM Tris-HCl (pH 7.6), 137 mM NaCl, and 0.1% Tween-20 for an hour before incubation at 4 °C overnight with the following primary antibodies: rabbit anti-cMYC (1:1000, Cell Signaling Technologies, Beverly, MA, USA), rabbit anti-PA2G4 (1:2000, Atlas Antibodies, Stockholm, Sweden, Cat# HPA016484), mouse anti-Vinculin (1:2000, Sigma-Aldrich, Cat# V9131), mouse anti-GAPDH (1:2000, Santa Cruz Biotechnology, Santa Cruz, CA, USA, Cat# sc-365062), and rabbit anti-β-actin (1:5000, Sigma, Cat# SAB2100037). Anti-mouse or anti-rabbit horseradish peroxidase secondary antibodies (1:5000, Life Technologies, Cat# 31430, 31460) were added and incubated for 2 h at room temperature. Immunoblots were visualized by Clarity ECL reagent (Bio-Rad) and ChemiDoc MP Imaging System (Bio-Rad). Quantification of protein expression was measured by Image Lab software v6.1 (Bio-Rad) and normalized to loading control.

### 2.4. siRNA Transfection

For siRNA-mediated knockdown, 40 nM of Qiagen (Millennium Science Australia, Victoria, Australia) custom PA2G4 duplex oligos (PA2G4 siRNA#1: 5′-GAGCAACAGGAGCAAACTATT-3′, PA2G4 siRNA#2: 5′-ACTGAGCCTGTGTGAGAAATT-3′, or cMYC siRNA#5: 5′-ATGCTATTGCTGTTCTAATTA-3′, cMYC siRNA #7 5′-GATGAGGAAGAAATCGATG-3′) were transfected with lipofectamine 2000 (Life Technologies) according to the manufacturer’s instructions. Dharmacon on-target plus control siRNA (Cat# D-001810-10-20) was used as siControl. Cells were transfected between 24, 48, 72, and 96 h, depending on the experimental requirements.

### 2.5. Animal Studies

All experimental procedures involving mice were approved by the University of New South Wales Animal Care and Ethics Committee according to the Animal Research Act, 1985 (Australia), and the Australian Code of Practice for Care and Use of Animals for Scientific Purposes 2015 (Approval number: 20/68B, Approval date: April 2020). The TH-MYCN transgenic mouse model of neuroblastoma used has been previously described [17]. To create PA2G4DelEx2-5 transgenic mice, double-stranded breaks were created within the PA2G4 locus (chromosome 10) in C57BL/6J mice using CRISPR/Cas9 and 2 sgRNAs (ggccggtcgcgccggccgac and ccatacccggctgtatggtc) targeting the region of exons 1–5 of the *PA2G4* coding sequence. To detect the wild-type allele, mice were genotyped using forward primer ATGTCATTAAGGCCGCTCAC and reverse primer GGCTGGGTACCTTCAAAGTG. The expected wildtype PCR product size is 471 bp. To detect the deleted allele, mice were genotyped using forward primer CAGCACCCTAGGACTTCCAC and reverse primer CACCAGGGAAGAAAAGATGAC. The expected deleted PCR product size is 374 bp. Hemizygous PA2G4^WT/DelEx1–5^ mice were crossed with Th-MYCN hemizygous transgenic mice and backcrossed to congenicity over three generations. Offspring were palpated twice a week and monitored for tumor development over a 52-week period. Mice were culled, necropsies were performed, and tumors were collected for IHC analysis once the tumor reached 1 cm^3^ or at the end of 52 weeks.

### 2.6. Single-Cell and Bulk-RNA Sequencing Analyses of Publicly Available Datasets

For single-cell neuroblastoma analysis, the publicly available NBAtlas dataset (https://data.mendeley.com/datasets/yhcf6787yp/3; accessed on 7 September 2025) [18] was utilized. Processed and annotated data were downloaded, and cells annotated as neuroendocrine were extracted. Patients with fewer than 100 neuroendocrine cells, or with fewer than 100 malignant or 100 normal neuroendocrine cells, were excluded. Normal adrenal medulla single-nucleus data were obtained from Jansky et al. (2021), and developmental adrenal medulla datasets (including 8 weeks postconception) were downloaded from https://adrenal.kitz-heidelberg.de/developmental_programs_NB_viz/; accessed on 7 September 2025) [19]. Pseudobulk profiles were generated at the patient level for NBAtlas by averaging expression across cells within malignant and normal neuroendocrine subsets. Spearman correlation analysis was performed at both the single-cell and pseudobulk levels using the correlatePairs function from the scater R package (v1.32.1), with *p*-values adjusted using the Benjamini–Hochberg method.

For bulk RNA-seq, TPM-normalized expression data for malignant and normal cell lines were obtained from the Cancer Cell Line Encyclopedia (CCLE) and Human Protein Atlas (HPA) (PMID: 37669926) via Zenodo (https://zenodo.org/records/7874749; accessed on 7 September 2025)) [20]. Immune cell datasets, comprising 29 sorted immune cell types from 4 individuals and PBMCs from 13 individuals, were obtained from GSE107011. For all bulk datasets, TPM-normalized counts were log_2_-transformed [log_2_(TPM + 1)] prior to analysis. Spearman correlation coefficients (ρ), *p*-values, and confidence intervals were calculated using GraphPad Prism 10.

### 2.7. Statistical Analysis

All statistical analysis was conducted using GraphPad Prism 10 software. Differences between groups were evaluated using unpaired two-sided *t*-tests. All data are presented as mean ± standard error of the mean (SEM) from at least three independent biological replicates.

## 3. Results

### 3.1. Loss of PA2G4 Reduces MYCN-Driven Neuroblastoma Tumorigenesis In Vivo

**Figure 1:**
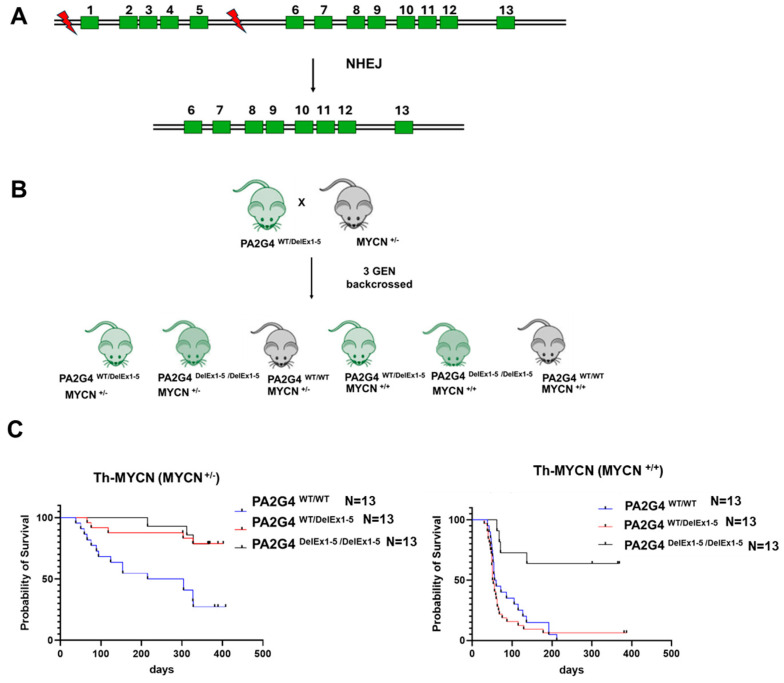
*PA2G4* gene knockout decreases neuroblastoma tumorigenesis in Th-MYCN transgenic mice. (**A**) *PA2G4* whole-body knockout mice were generated by deleting exons 1 to 5 of the *PA2G4* gene (located on mouse chromosome 10) using a double-stranded DNA break within the PA2G4 locus; (**B**) Hemizygous *PA2G4*^WT/DelEx1–5^ mice were backcrossed with Hemizygous Th-MYCN^+/−^ mice for three generations. (**C**) Kaplan–Meir Plot depicting proportion of hemizygous Th-*MYCN*^+/−^ (left panel) or Th-*MYCN*^+/+^ (right panel), crossed with either *PA2G* ^DelEx1-/DelEx1–5^ *PA2G4*^WT/DelEx1–5^ or *PA2G4*^WT/WT^, surviving tumor-free.

We previously demonstrated that pharmacological inhibition of the PA2G4-MYCN protein interaction using the small molecule WS6 exerts anti-tumorigenic effects in neuroblastoma models [12,14]. In vitro, *PA2G4* knockout partially reduced cell viability in neuroblastoma cell lines, with effects observed in MYCN-dependent and -independent contexts. To investigate the functional importance of PA2G4 in MYCN-driven neuroblastoma in vivo, we utilized the Th-*MYCN* transgenic mouse model, in which the human *MYCN* gene is expressed under the control of the tyrosine hydroxylase promoter. This model reliably develops neuroblastoma in 100% of homozygote animals by 6–8 weeks of age [16]. To determine the importance of PA2G4 to MYCN-driven neuroblastoma in vivo, we created Th-*MYCN* transgenic mice harboring a deletion of the *PA2G4* gene. A whole body *PA2G4* knockout mouse model was established using CRISPR/Cas-9 technology, introducing double-strand breaks within the *PA2G4* locus using sgRNAs targeting exons 1–5 of the *PA2G4* coding sequence in C57BL/6 mice (Figure 1A). Hemizygous *PA2G4*^WT/DelEx1–5^ mice were crossed with Th-MYCN hemizygous transgenic mice and bred to congenicity over three generations. This breeding strategy produced six genotypic groups: homozygous Th-*MYCN*^+/+^ crossed with either hemizygous *PA2G4*^WT/DelEx1–5^, homozygous *PA2G4* ^DelEx1-/DelEx1–5^ knockout mice, or *PA2G4* WT mice; and hemizygous Th-*MYCN*^+/−^ crossed with hemizygous *PA2G4*^WT/DelEx1–5^, homozygous *PA2G4* ^DelEx1-/DelEx1–5^ knockout mice, and *PA2G4* WT mice (Figure 1B). The *PA2G4* knockout mice showed no obvious phenotype. Immunohistochemical analysis of tumors from each genotype confirmed differential expression of PA2G4 and MYCN (Supplementary Figure S1).

The *PA2G4*^WT/WT^ x Th-*MYCN*^+/+^ mice exhibited 100% postnatal mortality due to aggressive neuroblastoma development (Figure 1C). Strikingly, homozygous deletion of *PA2G4* ^DelEx1-/DelEx1–5^ significantly increased the survival probability of homozygous Th-*MYCN*^+/+^ mice from 0% to 60%, whereas heterozygous deletion of *PA2G4*^WT/DelEx1–5^ had no significant effect on survival in this genotype. (Figure 1C). Hemizygous Th-*MYCN*^+/−^ mice had a survival probability of 30% when the *PA2G4* gene was wildtype, whereas both hemizygous *PA2G4*^WT/DelEx1–5^ and homozygous *PA2G4* ^DelEx1-/DelEx1–5^ knockout equally increased tumor-free survival probability of the homozygous Th-*MYCN*^+/+^ mice to 80% (Figure 1C). These findings demonstrate that PA2G4 is required for efficient MYCN-driven neuroblastoma tumorigenesis in vivo, and its loss impairs neuroblastoma progression in a gene dosage-dependent manner. When more MYCN is present, knockdown of both alleles of *PA2G4* is required for inhibition of tumor development.

To investigate correlations between PA2G4 and MYCN or MYC in the context of neuroblastoma and neuroendocrine development, we analyzed publicly available single-cell datasets from the neuroblastoma atlas (NBAtlas, Bonine et al., 2024 [18]) (Supplementary Figure S2) and the normal adrenal gland (Jansky et al., 2021 [19]) (Supplementary Figure S3). In MYCN-amplified malignant neuroendocrine cells, PA2G4 expression correlated strongly with MYCN (ρ = 0.65, *p* < 0.001) and moderately with MYC (ρ = 0.42, *p* < 0.001). This relationship persisted when restricting the analysis to cells co-expressing both genes (ρ = 0.70 and ρ = 0.49, respectively). In NBAtlas (Supplementary Figure S2), we observed a significant positive correlation between PA2G4 and MYCN in both malignant and normal neuroendocrine cells, with malignant cells showing slightly stronger correlations (Supplementary Figure S2d,e; left panels). While single-cell analyses suggested a correlation between PA2G4 and MYC, this did not hold at the pseudobulk level for either malignant or normal cells. Notably, MYC expression was relatively sparse across this dataset.

Taken together, these results suggest that in the NBAtlas dataset, where MYCN is more frequently expressed than MYC, PA2G4 shows a stronger correlation with MYCN. The relationship between PA2G4 and MYC remains less clear, particularly in contexts where MYC expression is low, and may depend on tumor subtype. This leaves open the possibility that in MYC-driven neuroblastoma, PA2G4 could also play a role, though further datasets will be needed to clarify this.

### 3.2. c-MYC Regulates PA2G4 Transcription

**Figure 2:**
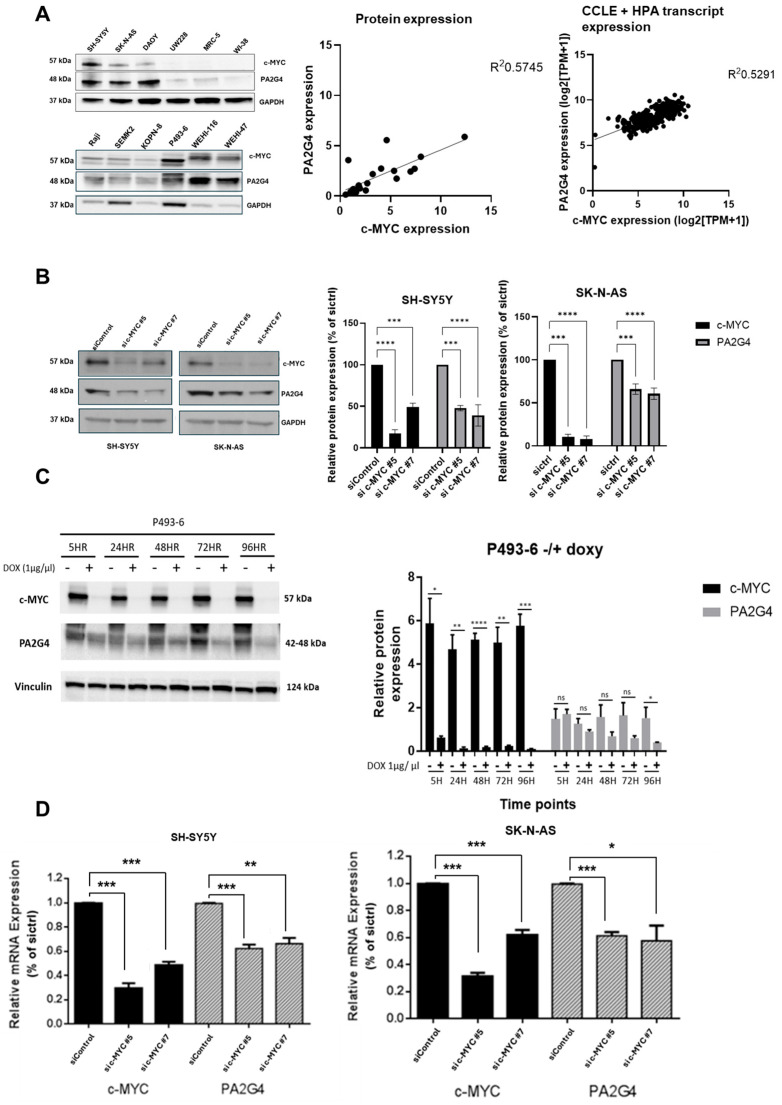
c-MYC regulates PA2G4 expression. (**A**) Western blot analysis showing PA2G4 and c-MYC protein expression levels in a panel of human neuroblastoma (SH-SY5Y, SK-N-AS), medulloblastoma (DAOY, UW-228), Burkitt lymphoma (Raji and P493-6), acute lymphoblastic leukemia (KOPN-8 and SEM-K2) and Murine B-cell ALL (WEHI-47 and WEHI-116) cell lines, and normal fibroblasts (MRC-5, WI-38) for a correlation between PA2G4 and c-MYC expression in the Western blot (r = 0.57) and CCLE + HPA transcript expression dataset (r = 0.53); (**B**) Western blot analysis showing PA2G4 and c-MYC protein expression levels after siRNA knockdown of c-MYC in SH-SY5Y cells and SK-N-AS cells at 48 h in comparison to siRNA control and using GAPDH as loading control; (**C**) Western blot analysis showing PA2G4 and c-MYC protein expression levels after Dox-inducible knockdown of c-MYC in P493-6 cells; (**D**) real-time PCR analysis of c-MYC and PA2G4 mRNA expression following c-MYC siRNA knockdown in SH-SY5Y and SK-N-AS human neuroblastoma cells. Statistical significance was determined using Student’s *t*-test. * *p* < 0.05, ** *p* < 0.01, *** *p* < 0.001, **** *p* < 0.0001, ns not significant.

Given the shared biological functions and 80% structural homology between MYCN and c-MYC [16], we investigated a potential regulatory relationship between c-MYC and PA2G4. Western blot analysis was performed to quantify basal levels of both c-MYC and PA2G4 proteins in a panel of cancer cell lines (Figure 2A), including neuroblastoma (SH-SY5Y, SK-N-AS), medulloblastoma (DAOY, UW-228), and Burkitt Lymphoma and B-Acute Lymphoblastic Leukemia (ALL) cell lines (Raji, KOPN-8, SEMK2, P493-6, WEHI-116, WEHI-47). All malignant cells expressed moderate to high levels of both c-MYC and PA2G4, except the medulloblastoma cell line, UW228. This was in contrast to normal fibroblasts (MRC-5, WI-38). Transcript expression of PA2G4 and c-MYC was analyzed in a panel of Neuroblastoma, Leukemia, Medulloblastoma, and non-cancerous cells using the publicly available datasets (CCLE, HPA) [20,21] (Supplementary Table S1), and a positive correlation (R^2^ = 0.57, R^2^ = 0.53) was observed between c-MYC and PA2G4 levels across these cell lines in both the Western blot and the transcript expression data (Figure 2A).

To determine whether c-MYC regulates PA2G4 protein expression, levels of PA2G4 were assessed by the immunoblotting of total protein extracts from human neuroblastoma cell lines (SH-SY5Y, SK-N-AS) 48 h after *c-MYC* siRNA knockdown. There was a significant (*p* < 0.01) decrease in PA2G4 protein expression after *c-MYC* knockdown in both cell lines (Figure 2B). Knockdown of *c-MYC* following the addition of Doxycycline (Dox) to the murine Burkitt lymphoma cell line, P493-6, in vitro also led to a significant (*p* < 0.05) decrease in c-MYC and PA2G4 protein expression over 96 h (Figure 2C). Real-time PCR analysis showed that *c-MYC* knockdown resulted in a significant (*p* < 0.01) decrease in *PA2G4* mRNA expression compared to the control after 48 h in neuroblastoma cells (Figure 2D), suggesting that c-MYC plays a role in PA2G4 transcriptional regulation.

### 3.3. PA2G4 Stabilizes c-MYC Protein

**Figure 3:**
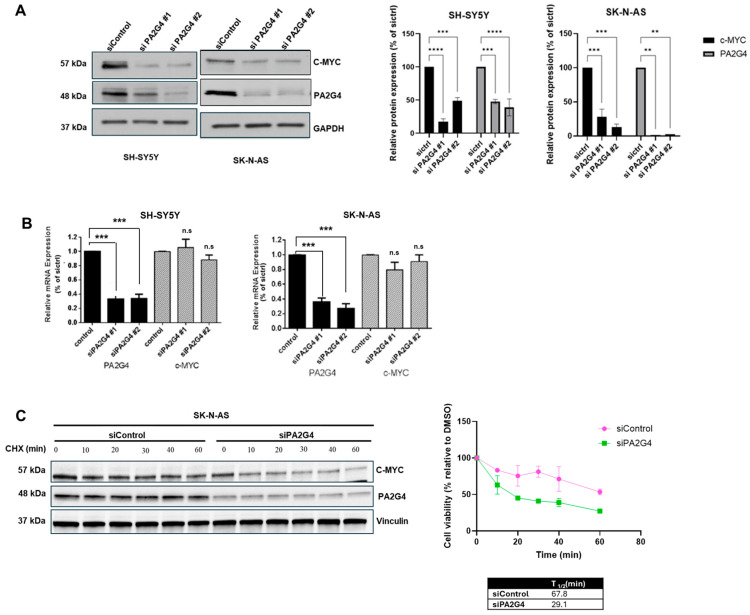
PA2G4 increases c-MYC protein stability. (**A**) Immunoblots showing PA2G4 and c-MYC protein expression levels after siRNA knockdown of PA2G4 in SH-SY5Y cells and SK-N-AS cells at 48 h and 96 h, respectively, compared to siControl. GAPDH is a loading control; (**B**) real-time PCR analysis of c-MYC and PA2G4 mRNA expression following PA2G4 siRNA knockdown in SH-SY5Y and SK-N-AS neuroblastoma cells; (**C**) knockdown with PA2G4 siRNA in SK-N-AS cells for 48 h, followed by treatment with 100 μg/μL cycloheximide for up to 60 min. Densitometry of c-MYC and PA2G4 protein levels on immunoblotting was used to estimate c-MYC protein half-life. Statistical significance was determined using Student’s *t*-test. ** *p* < 0.01, *** *p* < 0.001, **** *p* < 0.0001, n.s not significant.

To investigate whether PA2G4 forms a positive feed-forward expression loop with c-MYC, similar to its previously described interaction with MYCN [12], we transiently silenced *PA2G4* using siRNA in neuroblastoma cell lines. Protein and mRNA expressions of c-MYC and PA2G4 were examined using immunoblotting and qPCR, respectively. There was a significant decrease (*p* < 0.01) observed in both c-MYC and PA2G4 protein expression 48 h after PA2G4 knockdown in SH-SY5Y and SK-N-AS cells (Figure 3A). There was no change observed in *c-MYC* mRNA expression after *PA2G4* silencing (*p* > 0.05; Figure 3B). To further examine the effect of PA2G4 on c-MYC protein stability, a cycloheximide (CHX) chase assay was performed. PA2G4 expression was reduced in SK-N-AS cells transfected with a siRNA targeting *PA2G4* (siPA2G4#2) for 48 h. Addition of CHX (100 μg/μL) reduced protein expression of c-MYC in both siControl and si*PA2G4* conditions (Figure 3C); however, c-MYC degradation occurred more rapidly after *PA2G4* knockdown in comparison to control. The half-life of c-MYC protein decreased from 67.8 min to 29.1 min after PA2G4 knockdown (Figure 3C). Together, these results showed that PA2G4 was required for c-MYC protein stability in these neuroblastoma cell lines, independent of transcriptional regulation.

### 3.4. c-MYC Plays a ROLE in WS6-Induced Growth Inhibition in c-MYC-Driven Malignancies

**Figure 4:**
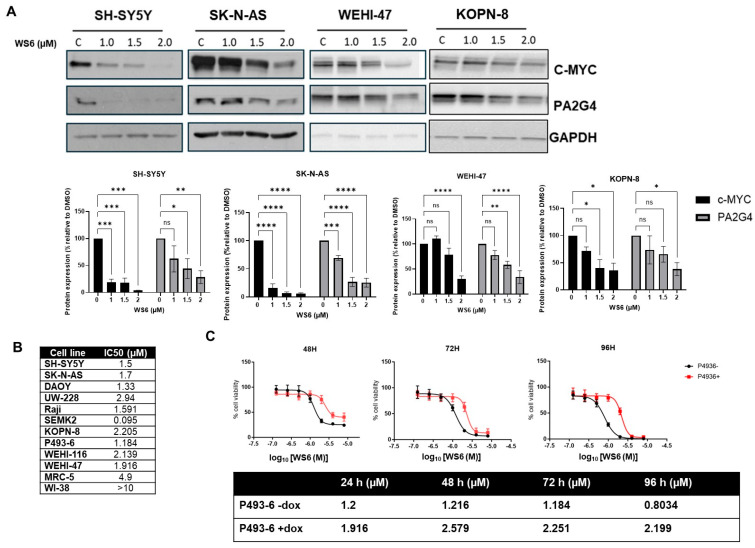
The growth inhibitory effects of WS6 on c-MYC-driven cancer cells. (**A**) Protein expression levels of PA2G4, c-MYC and loading control, GAPDH, in neuroblastoma (SH-SY5Y, SK-N-AS), B Lymphoma or ALL (WEHI47, KOPN-8) cancer cell lines treated with WS6, a small molecule inhibitor of PA2G4, for 72 h; (**B**) IC50 concentrations for WS6 in a panel of c-MYC-expressing cancer cell lines and normal fibroblasts after 72 h treatment; (**C**) cell viability and IC50 values of a c-MYC expressing murine lymphoma cell line with Doxycycline-regulable c-MYC knockdown (P493-6-dox: with c-MYC; and P493-6+dox: without c-MYC) following 48, 72, and 96 h of WS6 treatment (0–8 μM). Statistical significance was determined using Student’s *t*-test. * *p* < 0.05, ** *p* < 0.01, *** *p* < 0.001, **** *p* < 0.0001, ns not significant.

WS6 is a known small molecule inhibitor of PA2G4 [22]. Given our findings that PA2G4 and c-MYC form a positive forward feedback expression loop in MYC- and MYCN-driven cancers, we hypothesized that WS6 would repress the expression of both proteins in c-MYC-driven malignant cells. Neuroblastoma cell lines (SH-SY5Y and SK-N-AS and BL cell lines (WEHI-47 and KOPN-8), characterized by high c-MYC expression, were treated with increasing concentrations of WS6 (0–2 µM). Immunoblot analysis revealed a dose-dependent reduction in both c-MYC and PA2G4 protein levels in SK-N-AS and SH-SY5Y cells (Figure 4A) at all concentrations. Significant downregulation of these proteins in WEHI-47 and KOPN-8 lymphoma cells was only observed at the highest WS6 concentration (2 μM). WS6 demonstrated cytotoxic effects with IC50s (0.095–2.94 µM) in the neuroblastoma, medulloblastoma, leukemia, and lymphoma cell lines, compared to normal fibroblasts (4.9 & >10 µM) (Figure 4B and Figure S4).

To investigate whether c-MYC was necessary for the WS6-mediated cytotoxicity, we used the P493-6 Burkitt lymphoma cell line, which expresses a doxycycline-repressible c-MYC transgene [23]. WS6 was significantly more cytotoxic in c-MYC-expressing P493-6 -Dox cells, compared to P493-6 +Dox cells. The IC50 of WS6 increased to twice as much after the addition of Dox, with the greatest change observed in the IC50 at 96 h from 0.8 µM in c-MYC-expressing cells (P493-6 -dox) to 2.2 µM in c-MYC-suppressed cells (P493-6 +dox) (Figure 4C). These results indicate that c-MYC knockdown reduces sensitivity to WS6 treatment, thus demonstrating that the growth inhibitory effects of the PA2G4 inhibitor, WS6, are partially c-MYC dependent.

**Figure 5:**
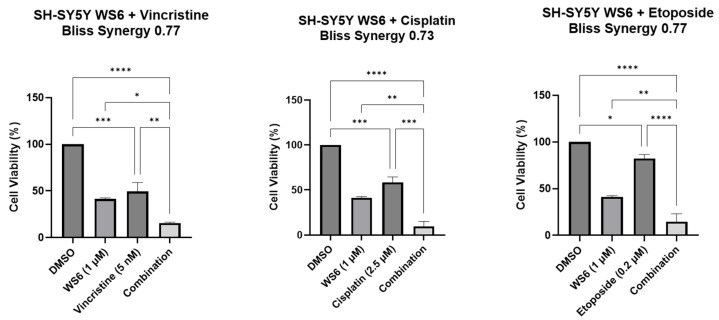
Combination therapy for neuroblastoma cells with WS6 and chemotherapeutic agents. Histogram depicting cell viability of SH-SY5Y cells treated with DMSO, WS6, Vincristine, Cisplatin, Etoposide, or WS6 + Vincristine, Cisplatin or Etoposide. Statistical significance was determined using Student’s t-test. * *p* < 0.05, ** *p* < 0.01, *** *p* < 0.001, **** *p* < 0.0001.

Given that cytotoxic chemotherapy is routinely used to treat each of these c-MYC-driven malignancies [24,25,26], we hypothesized that chemical PA2G4 and c-MYC inhibition by WS6 would have synergistic growth-inhibitory effects with chemotherapy in c-MYC-driven malignant cells. We examined the effects of WS6 used in combination with either Vincristine, Cisplatin, or Etoposide on the cell viability of the neuroblastoma cell line, SH-SY5Y. Synergy was determined using BLISS [27] (Supplementary Figure S5). The combinations of WS6 with these conventional chemotherapy agents (Vincristine, Cisplatin, and Etoposide) led to synergistic cytotoxicity when compared to the single agents alone (Figure 5).

## 4. Discussion

Our study provides novel insights into the role of PA2G4 as a critical regulator of c-MYC stability and underscores its potential as a therapeutic target across *MYC*-driven cancers. We present the first in vivo evidence demonstrating the essential role of PA2G4 in *MYCN*-driven neuroblastoma [12]. Mechanistically, we demonstrated that PA2G4 is essential for maintaining c-MYC protein stability and cell viability across various c-MYC-expressing cancer cell lines. Pharmacological inhibition of PA2G4 using the small molecule inhibitor WS6 resulted in a decrease in both c-MYC and PA2G4 protein levels and significantly enhanced cytotoxicity in c-MYC overexpressing cell lines. Together, these findings establish PA2G4 as a critical modulator of *MYC* oncogenic activity and a promising therapeutic vulnerability in c-MYC-driven malignancies.

Our study demonstrates for the first time that PA2G4 is essential for *MYCN*-driven tumorigenesis in vivo, as knockout of *PA2G4* significantly increased the survival of Th-*MYCN* mice. Notably, both alleles of *PA2G4* had to be knocked out to observe a survival benefit in homozygous Th-MYCN mice. However, in hemizygous Th-*MYCN* mice, a single allele knockout was sufficient to prolong survival, suggesting a gene dosage effect between *MYCN* and *PA2G4* in driving tumor progression.

MYC is an intrinsically disordered protein that has long been considered “undruggable” due to the absence of a defined binding pocket. As such, previous efforts have primarily focused on indirect strategies to target MYC, including disrupting the MYC/MAX dimer [28,29] or inhibiting its interaction with various other protein binding partners [30]. Compounds that target the MYC/MAX interaction have some effect in cancer, though these inhibitors often suffer from limited potency and significant off-target effects [31,32]. As a result, attention has shifted toward regulatory proteins that influence MYC stability. The stability of the MYC protein is tightly regulated through phosphorylation and interactions with specific proteins. Phosphorylation at Ser62 (by ERK/CDK) followed by Thr58 (by GSK3β) allows the E3 ubiquitin ligase FBXW7 to recognize and target MYC for degradation [9]. However, this process can be counteracted. PP2A stabilizes MYC by dephosphorylating Ser62 [9]. PIN1 prevents FBXW7 recognition by isomerizing the phosphorylated Thr58-Pro motif [7]; and deubiquitinases USP28 and USP7 [8], as well as Aurora A kinase, protect MYC from degradation [33,34]. Our study introduces PA2G4 as a novel regulator of MYC protein stability. We demonstrate that PA2G4 stabilizes c-MYC in cancer cells, thereby expanding the landscape of MYC-interacting proteins that can be exploited for therapeutic intervention. Targeting PA2G4 may offer a new and more selective approach to destabilize MYC in malignancies where direct targeting remains challenging.

PA2G4 is a DNA and RNA-binding protein involved in key cellular processes such as growth, apoptosis, and differentiation [33,34,35], with the longer isoform, PA2G4-p48, exhibiting oncogenic properties [33]. Overexpression of PA2G4 has been associated with enhanced tumor progression in glioblastoma and oral squamous cell carcinoma pre-clinical models [36], supporting its role as a driver of malignancy. In line with these findings, a recent proteogenomic analysis identified the PA2G4-MYC axis as a druggable vulnerability in 3q26-amplified acute myeloid leukemia (AML) [22], where PA2G4 overexpression conferred resistance to HDAC inhibitors, while its inhibition suppressed leukemia progression in patient-derived xenograft models. Our study builds on this emerging evidence and further supports PA2G4 as an oncogenic cofactor in MYC-driven cancers. We show that PA2G4 plays a critical role in stabilizing MYC proteins, reinforcing its function as a MYC family co-regulator. Previous studies have shown that PA2G4 interacts with FBXW7α, sequestering it in the cytoplasm and thereby preventing it from targeting MYC for proteasomal degradation [29]. This interaction effectively prolongs the half-life of MYC proteins, enhancing their oncogenic potential. This interaction leads to increased MYC protein stability and sustained oncogenic signaling. By shielding MYC from degradation, PA2G4 amplifies MYC-driven transcriptional programs and tumorigenic potential. Taken together with prior studies, our findings in neuroblastoma highlight PA2G4 as a conserved and functionally significant MYC regulatory factor which may have a role in many c-MYC-driven malignancies. Targeting the PA2G4–MYC axis represents a promising therapeutic strategy for MYC-driven cancers.

One promising therapeutic avenue is the use of PA2G4 inhibition to enhance the efficacy of conventional chemotherapies. In cancers where MYC plays a critical role in tumor growth and chemoresistance, combining PA2G4 inhibition with chemotherapy could provide a more effective strategy for overcoming drug resistance and improving patient outcomes. Additionally, the potential synergy between PA2G4 inhibitors and other targeted therapies, such as those aimed at MYC-interacting proteins like FBXW7 or Aurora A, could further enhance the therapeutic benefit. These combinatorial approaches warrant further investigation as a strategy to exploit MYC-driven vulnerabilities and broaden treatment options for patients with high-risk malignancies.

While our findings highlight the therapeutic potential of targeting PA2G4 in *MYC*-driven cancers, several important limitations remain. Although we demonstrated that PA2G4 is essential for maintaining c-MYC stability and function in vitro, the in vivo efficacy of PA2G4 inhibition in human c-MYC-driven tumors has yet to be fully established. Previous studies have shown that WS6 is effective in MYCN-driven neuroblastoma models; however, its activity in other *MYC*-driven cancers remains to be determined. Moreover, we observed that WS6-induced cytotoxicity varied across cancer cell lines, suggesting the possibility of off-target effects or cell line-specific differences in dependency on PA2G4. These findings underscore the need to further characterize the broader network of PA2G4 interactions, particularly with proteins involved in MYC regulation, to better understand its context-specific roles in oncogenesis. A major limitation of WS6 as a therapeutic agent is its known toxicity in normal tissues, which significantly restricts its translational potential. To overcome this, our previous work focused on developing and characterizing WS6 analogs with improved selectivity, reduced toxicity, and enhanced drug-like properties [16]. These efforts aim to generate more clinically viable PA2G4 inhibitors for future therapeutic application. To further advance this therapeutic strategy, structural studies of the PA2G4–MYC complex will be crucial. Future studies will focus on elucidating the precise mechanism by which PA2G4 regulates MYC stability, including evaluation of MYC phosphorylation status and its regulation via the proteasome pathway in PA2G4-deficient and -proficient contexts across multiple cancer types. High-resolution structural analyses could reveal key binding sites and conformational changes that occur upon interaction, providing a blueprint for rational drug design. Ultimately, understanding the structural basis of *PA2G4*′s oncogenic function will be essential for developing potent and selective inhibitors that disrupt its role in cancer while minimizing effects on its physiological functions.

## 5. Conclusions

In summary, our study identifies PA2G4 as a novel and critical regulator of MYC protein stability, establishing it as a promising therapeutic target in MYC-driven cancers. By demonstrating that disruption of PA2G4 impairs MYC stability and tumor cell viability, we provide a compelling rationale for targeting the PA2G4–MYC axis as an alternative to direct MYC inhibition. These findings lay the groundwork for future development of clinically viable PA2G4 inhibitors and open new avenues for the treatment of high-risk, MYC-driven malignancies.

## Data Availability

For single-cell neuroblastoma analysis, the publicly available NBAtlas dataset (https://data.mendeley.com/datasets/yhcf6787yp/3; accessed on 7 September 2025) [18] was utilized. Normal adrenal medulla single-nucleus data were obtained from Jansky et al. (2021) [19], and developmental adrenal medulla datasets (including 8 weeks postconception) were downloaded from https://adrenal.kitz-heidelberg.de/developmental_programs_NB_viz/; accessed on 7 September 2025. For bulk RNA-seq, TPM-normalized expression data for malignant and normal cell lines were obtained from the Cancer Cell Line Encyclopedia (CCLE) and Human Protein Atlas (HPA) (PMID: 37669926) via Zenodo (https://zenodo.org/records/7874749; accessed on 7 September 2025). Data underlying all figures and supplementary figures, uncropped and unprocessed immunoblot scans, are provided. All other relevant data are available from the corresponding authors on request.

## Supplementary Materials

The following supporting information can be downloaded at: https://www.mdpi.com/article/10.3390/cells14181422/s1, Figure S1: Immunohistochemical analysis of MYCN and PA2G4 in MYCN x PA2G4 mice; Figure S2: Single-cell RNA sequencing analysis of the NBatlas; Figure S3: Single-cell RNA sequencing analysis of the adrenal medulla datasets; Figure S4: Cell viability after treatment with WS6. Figure S5: BLISS synergy maps for WS6 combination treatment; Table S1: CCLE + HPA transcript expression cell line data.
