# Supplementary material for: PA2G4 Functions as a Cofactor for MYC Family Oncoproteins in MYC-Driven Malignancies
Source: Cells. 2025 Sep 11;14(18):1422. doi: 10.3390/cells14181422 (PMC12468391; doi:10.3390/cells14181422)
Supplement: Supplementary file 1 [file cells-14-01422-s001.zip › cells-3789069-supplementary figures.pptx]

## Slide 1
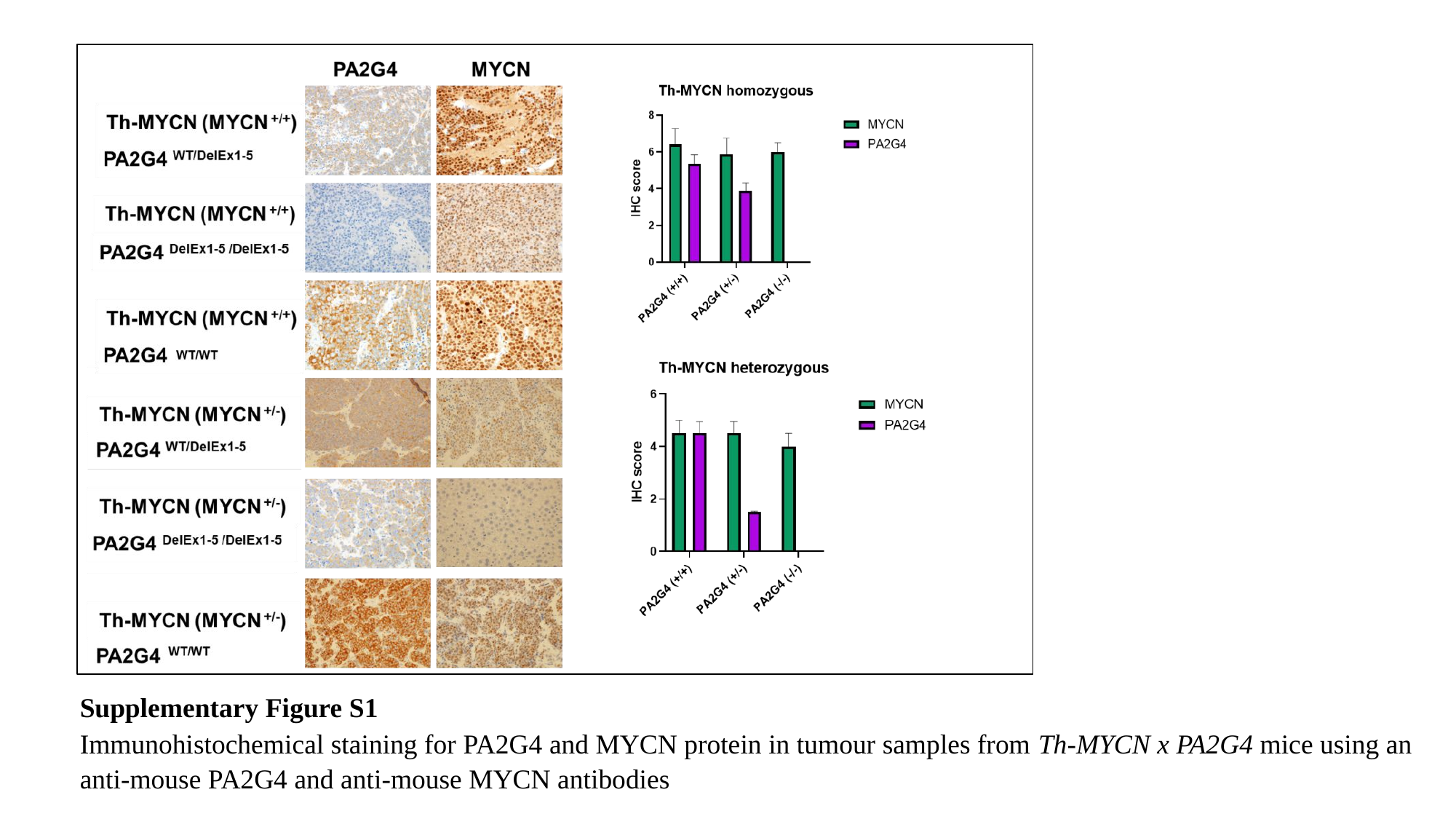

Supplementary Figure S1
Immunohistochemical staining for PA2G4 and MYCN protein in tumour samples from Th-MYCN x PA2G4 mice using an anti-mouse PA2G4 and anti-mouse MYCN antibodies

## Slide 2
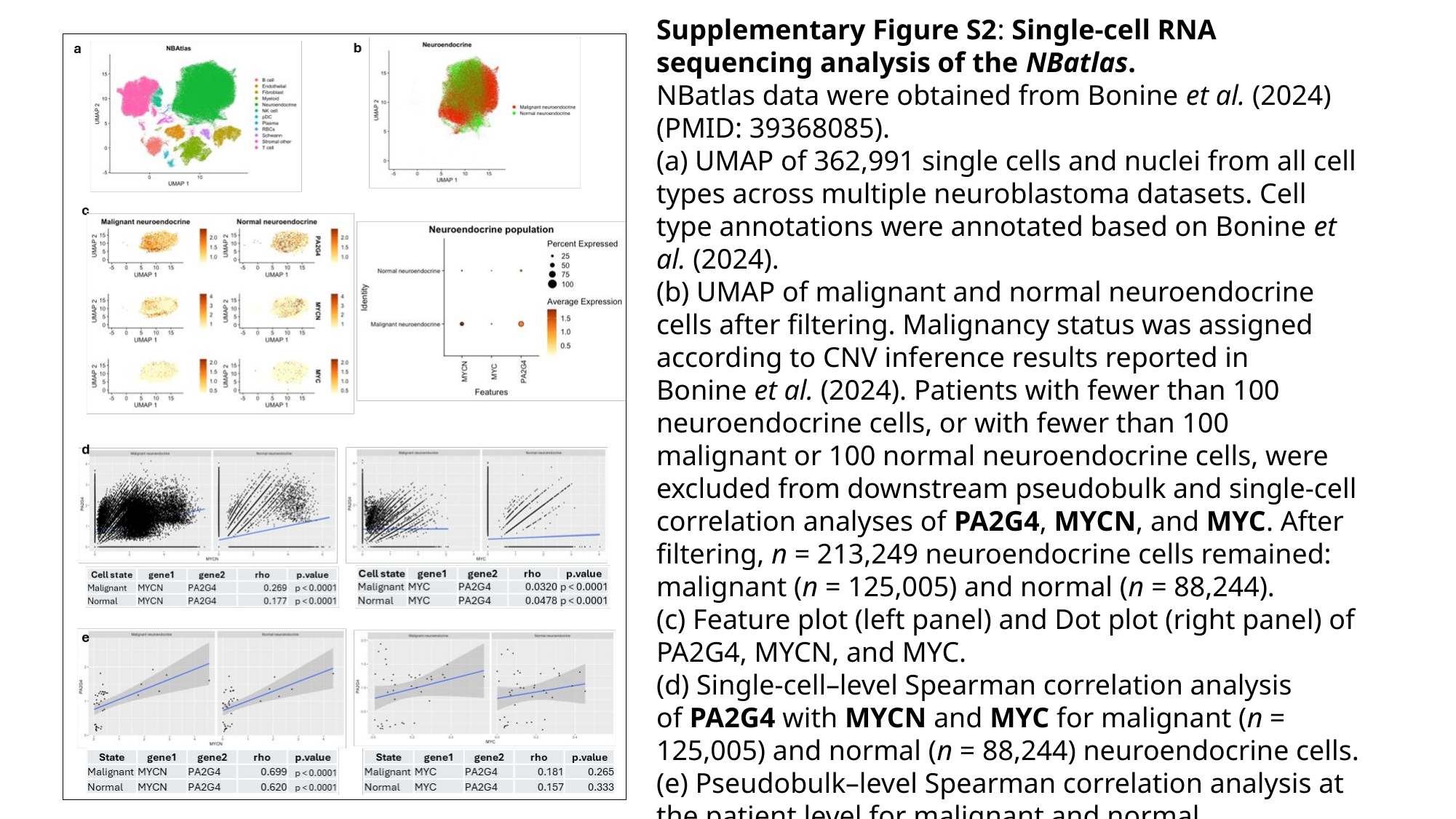

Supplementary Figure S2: Single-cell RNA sequencing analysis of the NBatlas.
NBatlas data were obtained from Bonine et al. (2024) (PMID: 39368085).
(a) UMAP of 362,991 single cells and nuclei from all cell types across multiple neuroblastoma datasets. Cell type annotations were annotated based on Bonine et al. (2024).
(b) UMAP of malignant and normal neuroendocrine cells after filtering. Malignancy status was assigned according to CNV inference results reported in Bonine et al. (2024). Patients with fewer than 100 neuroendocrine cells, or with fewer than 100 malignant or 100 normal neuroendocrine cells, were excluded from downstream pseudobulk and single-cell correlation analyses of PA2G4, MYCN, and MYC. After filtering, n = 213,249 neuroendocrine cells remained: malignant (n = 125,005) and normal (n = 88,244).
(c) Feature plot (left panel) and Dot plot (right panel) of PA2G4, MYCN, and MYC.
(d) Single-cell–level Spearman correlation analysis of PA2G4 with MYCN and MYC for malignant (n = 125,005) and normal (n = 88,244) neuroendocrine cells.
(e) Pseudobulk–level Spearman correlation analysis at the patient level for malignant and normal neuroendocrine cells (n = x patients).

## Slide 3
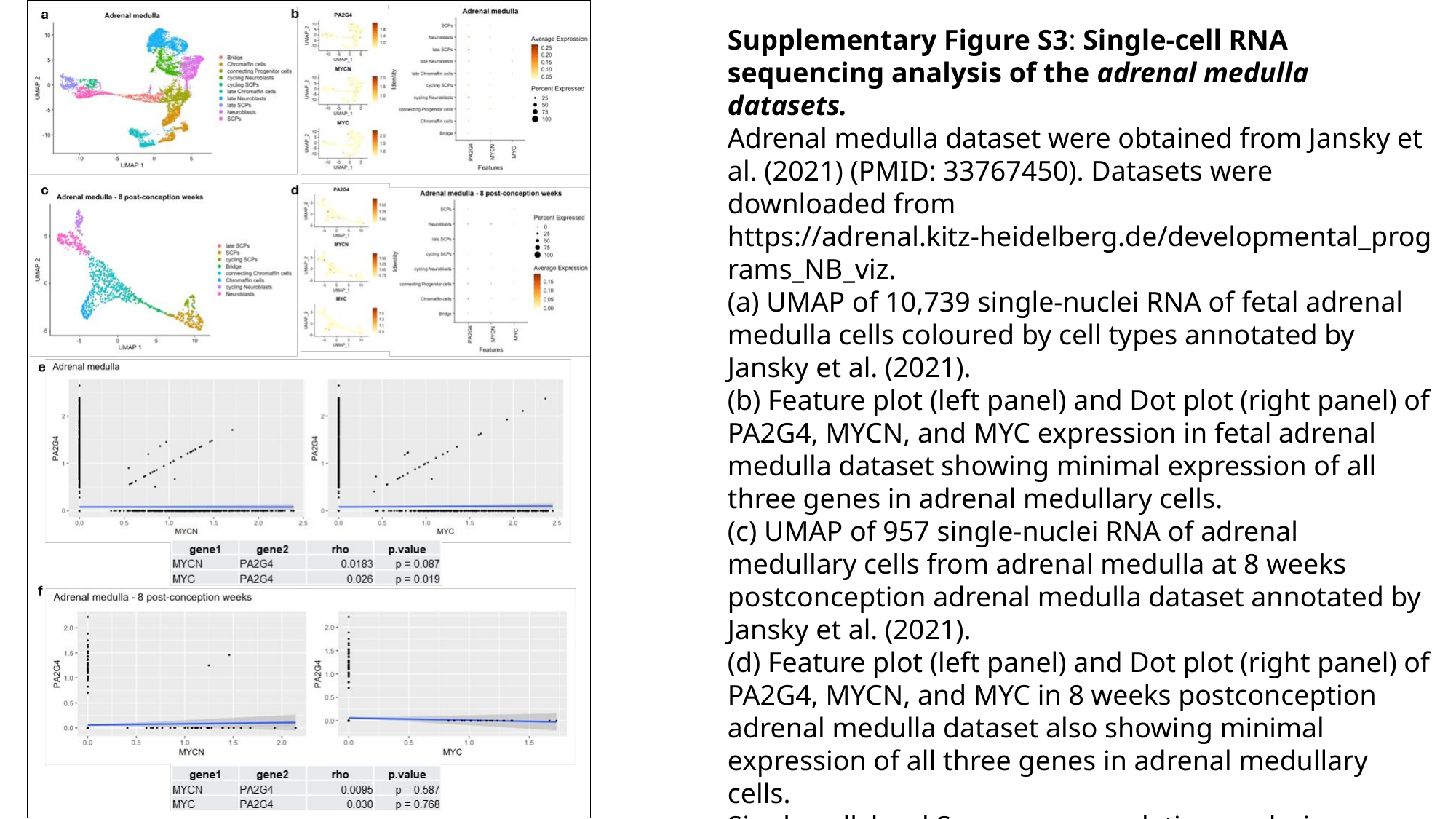

Supplementary Figure S3: Single-cell RNA sequencing analysis of the adrenal medulla datasets.
Adrenal medulla dataset were obtained from Jansky et al. (2021) (PMID: 33767450). Datasets were downloaded from https://adrenal.kitz-heidelberg.de/developmental_programs_NB_viz.
(a) UMAP of 10,739 single-nuclei RNA of fetal adrenal medulla cells coloured by cell types annotated by Jansky et al. (2021).
(b) Feature plot (left panel) and Dot plot (right panel) of PA2G4, MYCN, and MYC expression in fetal adrenal medulla dataset showing minimal expression of all three genes in adrenal medullary cells.
(c) UMAP of 957 single-nuclei RNA of adrenal medullary cells from adrenal medulla at 8 weeks postconception adrenal medulla dataset annotated by Jansky et al. (2021).
(d) Feature plot (left panel) and Dot plot (right panel) of PA2G4, MYCN, and MYC in 8 weeks postconception adrenal medulla dataset also showing minimal expression of all three genes in adrenal medullary cells.
Single-cell–level Spearman correlation analysis of PA2G4 with MYCN and MYC for (e) fetal adrenal medulla and (f) 8 PCW adrenal medulla datasets.

## Slide 4
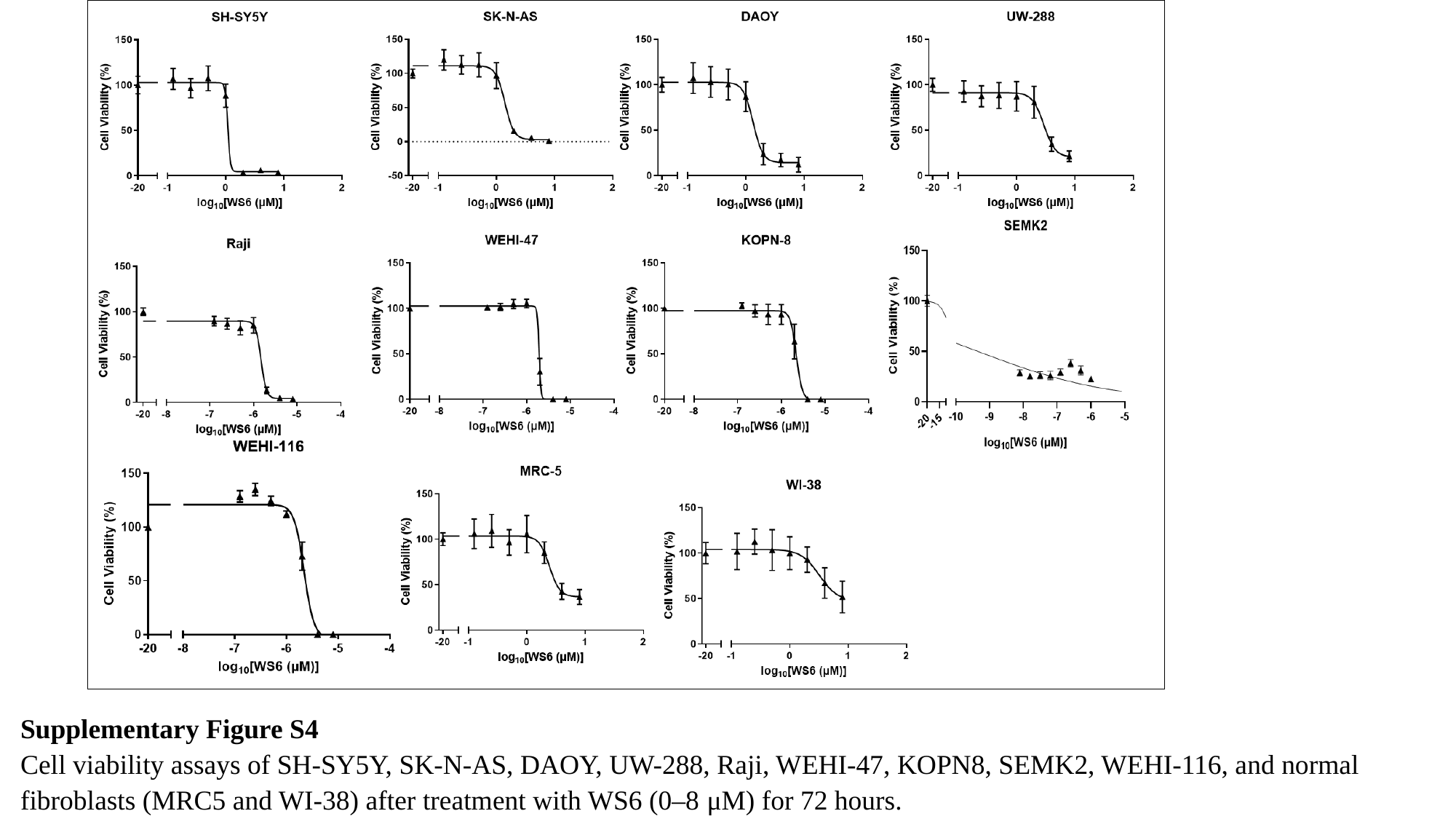

Supplementary Figure S4
Cell viability assays of SH-SY5Y, SK-N-AS, DAOY, UW-288, Raji, WEHI-47, KOPN8, SEMK2, WEHI-116, and normal fibroblasts (MRC5 and WI-38) after treatment with WS6 (0–8 μM) for 72 hours.

## Slide 5
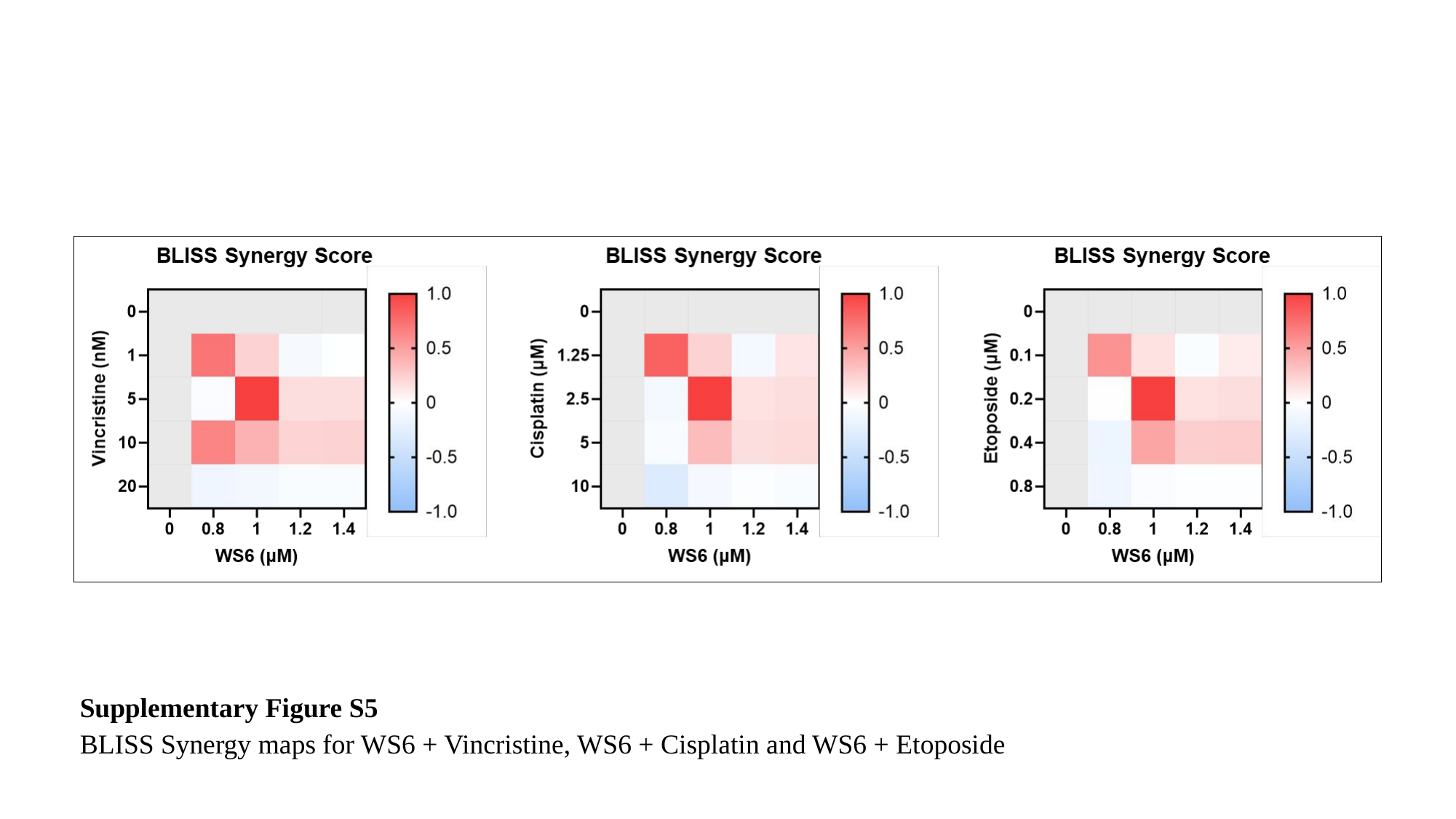

Supplementary Figure S5
BLISS Synergy maps for WS6 + Vincristine, WS6 + Cisplatin and WS6 + Etoposide
