# Supplementary material for: PA2G4 Functions as a Cofactor for MYC Family Oncoproteins in MYC-Driven Malignancies
Source: Cells. 2025 Sep 11;14(18):1422. doi: 10.3390/cells14181422 (PMC12468391; doi:10.3390/cells14181422)
Supplement: Supplementary file 1 [file cells-14-01422-s001.zip › Supplemtary table S1.pdf]

| sample | context | dataset | mary_disea | e_collectio | MYCN_log | MYC_log | PA2G4_log |
|---|---|---|---|---|---|---|---|
| CVCL_2162 | PLB-985 | CellLine | Leukemia | Haematopo | 0.080054157 | 10.51466748 | 9.332208649 |
| CVCL_3043 | NCO2 | CellLine | Leukemia | Haematopo | 1.43774905 | 10.07248217 | 8.863354445 |
| CVCL_0181 | BV-173 | CellLine | Leukemia | Haematopo | 0.098398828 | 9.996310883 | 9.612371363 |
| CVCL_2122 | MOLM-6 | CellLine | Leukemia | Haematopo | 4.326371659 | 9.780635446 | 9.014454417 |
| CVCL_0002 | HL-60 | CellLine | Leukemia | Haematopo | 0.037832588 | 9.706913783 | 9.650963805 |
| CVCL_1196 | EM-2 | CellLine | Leukemia | Bone Marr | 0.053934069 | 9.684155165 | 9.272275114 |
| CVCL_0258 | EoL-1 | CellLine | Leukemia | Haematopo | 0.059439043 | 9.547942725 | 9.076371164 |
| CVCL_2095 | KYO-1 | CellLine | Leukemia | Haematopo | 0.461013406 | 9.461674649 | 8.848386238 |
| CVCL_2106 | M-07e | CellLine | Leukemia | Haematopo | 3.553667434 | 9.322684466 | 9.136921481 |
| CVCL_0005 | NB4 | CellLine | Leukemia | Bone Marr | 0.19379634 | 9.27040496 | 9.40902391 |
| CVCL_0379 | Ku812 | CellLine | Leukemia | Haematopo | 0.474369768 | 9.217833405 | 8.93401073 |
| CVCL_2091 | KCL-22 | CellLine | Leukemia | Pleural Effu | 0.363029453 | 9.101257669 | 8.645443212 |
| CVCL_2481 | HEL 92.1.7 | CellLine | Leukemia | Bone Marr | 3.540320835 | 9.077738077 | 9.00667689 |
| CVCL_0589 | Kasumi-1 | CellLine | Leukemia | Haematopo | 3.09754598 | 9.074148403 | 8.967173466 |
| CVCL_2037 | F-36P | CellLine | Leukemia | Pleural Effu | 0.02191691 | 9.018123562 | 8.40959943 |
| CVCL_0388 | LAMA-84 | CellLine | Leukemia | Haematopo | 0.635874959 | 9.011914717 | 8.83179495 |
| CVCL_1424 | MOLT-16 | CellLine | Leukemia | Haematopo | 0.105252992 | 8.96736864 | 8.329043669 |
| CVCL_0374 | KG-1 | CellLine | Leukemia | Bone Marr | 5.131603714 | 8.862478994 | 7.949086401 |
| CVCL_0425 | MEG-01 | CellLine | Leukemia | Bone Marr | 0.388847349 | 8.859809123 | 9.302054443 |
| CVCL_0092 | NALM-6 | CellLine | Leukemia | Haematopo | 0.013419803 | 8.820794794 | 8.540235647 |
| CVCL_0001 | HEL | CellLine | Leukemia | Haematopo | 3.342157851 | 8.802047297 | 9.268329976 |
| CVCL_2119 | MOLM-13 | CellLine | Leukemia | Haematopo | 0.033940174 | 8.799932783 | 9.198349172 |
| CVCL_2149 | OCI-M1 | CellLine | Leukemia | Haematopo | 0.071657821 | 8.772899919 | 8.156814826 |
| CVCL_1409 | MHH-CALL- | CellLine | Leukemia | Haematopo | 0.133908647 | 8.711505186 | 8.905213221 |
| CVCL_1620 | OCI-AML-5 | CellLine | Leukemia | Haematopo | 0 | 8.690239322 | 8.933732521 |
| CVCL_1805 | ALL-SIL | CellLine | Leukemia | Haematopo | 0 | 8.688899513 | 9.249513426 |
| CVCL_0559 | TF-1 | CellLine | Leukemia | Bone Marr | 0.404903013 | 8.638538263 | 9.04681782 |
| CVCL_0013 | MOLT-4 | CellLine | Leukemia | haematopo | 0.533105799 | 8.627526219 | 9.455080813 |
| CVCL_Y019 | HAP1 | CellLine | Leukemia | bone_marr | 1.575465548 | 8.588244396 | 9.168149518 |
| CVCL_0098 | SKM-1 | CellLine | Leukemia | Haematopo | 0.545146649 | 8.587383156 | 8.265549124 |
| CVCL_2196 | SKNO-1 | CellLine | Leukemia | Bone Marr | 0.825784616 | 8.585837015 | 9.476323977 |
| CVCL_2120 | MOLM-16 | CellLine | Leukemia | Haematopo | 0.12522676 | 8.580446801 | 9.204338571 |
| CVCL_0064 | MV4-11 | CellLine | Leukemia | Haematopo | 0.080782345 | 8.573905607 | 9.139595408 |
| CVCL_1873 | MUTZ-5 | CellLine | Leukemia | Haematopo | 0.286629713 | 8.534483548 | 8.97363007 |
| CVCL_0216 | CMK | CellLine | Leukemia | Haematopo | 2.171324983 | 8.473580136 | 8.453935944 |
| CVCL_0091 | NALM-1 | CellLine | Leukemia | Haematopo | 0.14936012 | 8.469293069 | 8.787959853 |
| CVCL_2086 | JURL-MK1 | CellLine | Leukemia | Haematopo | 0.217579996 | 8.447387752 | 8.85197372 |
| CVCL_1071 | AML-193 | CellLine | Leukemia | Haematopo | 0.059408297 | 8.427199403 | 9.025706777 |
| CVCL_0004 | K-562 | CellLine | Leukemia | Pleural Effu | 0.24971288 | 8.3910751 | 9.206501799 |
| CVCL_2079 | JK-1 | CellLine | Leukemia | Haematopo | 0.432720703 | 8.386230536 | 9.524377421 |
| CVCL_1321 | KO52 | CellLine | Leukemia | Bone Marr | 5.871046155 | 8.371758355 | 8.744034437 |
| CVCL_1820 | HPB-ALL | CellLine | Leukemia | Haematopo | 0.095764197 | 8.365794266 | 8.881856486 |
| CVCL_1425 | Mono-Mac | CellLine | Leukemia | Haematopo | 0.023968125 | 8.359142056 | 8.346992035 |
| CVCL_0079 | 697 | CellLine | Leukemia | Bone Marr | 0.037306963 | 8.349093221 | 9.485734596 |
| CVCL_1619 | OCI-AML-2 | CellLine | Leukemia | Haematopo | 0.020435859 | 8.336904012 | 8.568973424 |
| CVCL_1844 | OCI-AML-3 | CellLine | Leukemia | Haematopo | 0 | 8.301802515 | 9.041371742 |
| CVCL_1851 | RCH-ACV | CellLine | Leukemia | Bone Marr | 0.423132895 | 8.300000141 | 9.653089492 |

| | | | | | | | |
|---|---|---|---|---|---|---|---|
| CVCL_1694 | SIG-M5 | CellLine | Leukemia | Bone Marr | 0.445972117 | 8.299356948 | 9.532796088 |
| CVCL_1327 | KE-37 | CellLine | Leukemia | Haematopo | 2.573862078 | 8.294154055 | 8.348260414 |
| CVCL_2187 | SET-2 | CellLine | Leukemia | Haematopo | 0.185769694 | 8.234045223 | 8.091007175 |
| CVCL_0007 | U-937 | CellLine | Leukemia | Pleural Effu | 0.054208443 | 8.229452942 | 8.941882208 |
| CVCL_1426 | Mono-Mac | CellLine | Leukemia | Haematopo | 0 | 8.0745156 | 8.753088847 |
| CVCL_3532 | JM-1 | CellLine | Leukemia | Haematopo | 0.00930261 | 8.059236022 | 10.15679422 |
| CVCL_0093 | RS4;11 | CellLine | Leukemia | Bone Marr | 0.021678077 | 7.952917307 | 9.27899391 |
| CVCL_1126 | CML-T1 | CellLine | Leukemia | Haematopo | 0.489686466 | 7.94114655 | 8.308068287 |
| CVCL_1714 | SUP-T1 | CellLine | Leukemia | Pleural Effu | 0 | 7.892520937 | 9.106915102 |
| CVCL_2022 | DND-41 | CellLine | Leukemia | Haematopo | 0 | 7.856441776 | 8.678847479 |
| CVCL_1870 | MEC-1 | CellLine | Leukemia | Haematopo | 0.041965736 | 7.774447133 | 9.103347976 |
| CVCL_1632 | P31/FUJ | CellLine | Leukemia | Haematopo | 0.048229733 | 7.766780632 | 8.24162968 |
| CVCL_1866 | KOPN-8 | CellLine | Leukemia | Haematopo | 0.231361542 | 7.753648335 | 9.62857234 |
| CVCL_1320 | JVM-3 | CellLine | Leukemia | Haematopo | 0.214699181 | 7.716807449 | 8.792651662 |
| CVCL_0095 | SEM | CellLine | Leukemia | Haematopo | 0.013934775 | 7.715877929 | 9.08101482 |
| CVCL_1410 | MHH-CALL- | CellLine | Leukemia | Haematopo | 0.18029772 | 7.695069333 | 8.940546315 |
| CVCL_1650 | Reh | CellLine | Leukemia | Haematopo | 0.139500267 | 7.67882701 | 9.283972687 |
| CVCL_1609 | NOMO-1 | CellLine | Leukemia | Bone Marr | 0.054428207 | 7.662620634 | 8.800534947 |
| CVCL_0624 | MOLT-3 | CellLine | Leukemia | Haematopo | 0.396321447 | 7.612585612 | 8.05664855 |
| CVCL_4613 | BDCM | CellLine | Leukemia | Haematopo | 0.136528043 | 7.499660355 | 9.303410308 |
| CVCL_0006 | THP-1 | CellLine | Leukemia | Haematopo | 0.032775753 | 7.482893674 | 8.52917183 |
| CVCL_1835 | NALM-19 | CellLine | Leukemia | Haematopo | 0.104736388 | 7.474606864 | 9.125200703 |
| CVCL_2071 | HNT-34 | CellLine | Leukemia | Haematopo | 5.555079675 | 7.473862167 | 8.648364934 |
| CVCL_1630 | P12-Ichikav | CellLine | Leukemia | Haematopo | 2.991609097 | 7.457751894 | 8.998329242 |
| CVCL_0065 | Jurkat | CellLine | Leukemia | Haematopo | 1.057112545 | 7.408621854 | 7.931521793 |
| CVCL_0089 | MHH-CALL- | CellLine | Leukemia | Bone Marr | 0.119796397 | 7.344323137 | 8.612106 |
| CVCL_2210 | SUP-T11 | CellLine | Leukemia | Bone Marr | 0 | 7.22009264 | 8.023713315 |
| CVCL_2161 | PL-21 | CellLine | Leukemia | Haematopo | 0.470270399 | 7.219739926 | 8.248208762 |
| CVCL_0590 | Kasumi-2 | CellLine | Leukemia | Bone Marr | 0.43078002 | 7.144188309 | 8.677927068 |
| CVCL_1422 | MOLT-13 | CellLine | Leukemia | Bone Marr | 3.808756822 | 7.020990611 | 8.362340991 |
| CVCL_1913 | Peer | CellLine | Leukemia | Haematopo | 0.34970002 | 6.96770458 | 9.313283048 |
| CVCL_0103 | SUP-B15 | CellLine | Leukemia | Bone Marr | 0.074019539 | 6.900694111 | 9.037536648 |
| CVCL_1736 | TALL-1 [Hu | CellLine | Leukemia | Bone Marr | 0 | 6.264850481 | 7.994741658 |
| CVCL_0003 | HMC-1 | CellLine | Leukemia | | 5.866808264 | 5.729212449 | 7.86702912 |
| CVCL_1641 | PF-382 | CellLine | Leukemia | Pleural Effu | 1.31632846 | 5.564857826 | 7.625885354 |
| CVCL_1380 | Loucy | CellLine | Leukemia | Haematopo | 5.222365187 | 0.941692369 | 8.219127825 |
| CVCL_2676 | P3HR-1 | CellLine | Lymphoma | Ascites | 0.121752314 | 9.816488957 | 10.28567093 |
| CVCL_1878 | OCI-Ly19 | CellLine | Lymphoma | Bone Marr | 0.279932107 | 9.762362881 | 9.275801883 |
| CVCL_1062 | A3/Kawaka | CellLine | Lymphoma | Ascites | 0.059959018 | 9.564236841 | 8.430507865 |
| CVCL_1876 | NU-DHL-1 | CellLine | Lymphoma | Lymph Noc | 0.294624797 | 9.462918749 | 9.914523111 |
| CVCL_0511 | Raji | CellLine | Lymphoma | Bone | 0.425515195 | 9.308008254 | 9.764094023 |
| CVCL_1877 | NU-DUL-1 | CellLine | Lymphoma | Central Ne | 0.195234161 | 9.079298505 | 9.222947864 |
| CVCL_1884 | REC-1 | CellLine | Lymphoma | Lymph Noc | 0.356447549 | 9.034152953 | 10.55112594 |
| CVCL_2207 | SU-DHL-8 | CellLine | Lymphoma | Pleural Effu | 0.019698082 | 8.940533212 | 9.731741922 |
| CVCL_1168 | DB | CellLine | Lymphoma | Ascites | 0.160009199 | 8.940411247 | 8.676301435 |
| CVCL_1712 | ST486 | CellLine | Lymphoma | Ascites | 0.061416653 | 8.924351271 | 8.734489597 |
| CVCL_1865 | JeKo-1 | CellLine | Lymphoma | Haematopo | 0.454545967 | 8.823970713 | 8.633361671 |
| CVCL_1399 | MC116 | CellLine | Lymphoma | Pleural Effu | 0.328732088 | 8.797019388 | 9.547972041 |

| | | | | | | | |
|---|---|---|---|---|---|---|---|
| CVCL_1861 | Ci-1 | CellLine | Lymphoma | Ascites | 0.131305794 | 8.794403455 | 9.780358616 |
| CVCL_0008 | Daudi | CellLine | Lymphoma | Haematopo | 0.0673046 | 8.783990466 | 9.243599823 |
| CVCL_1660 | RL | CellLine | Lymphoma | Ascites | 0.098240122 | 8.570842662 | 9.835108985 |
| CVCL_0539 | SU-DHL-4 | CellLine | Lymphoma | Pleural Effu | 0 | 8.569949953 | 9.333933438 |
| CVCL_0067 | Namalwa | CellLine | Lymphoma | Haematopo | 0.144478537 | 8.568254148 | 9.222502671 |
| CVCL_1222 | GA-10 | CellLine | Lymphoma | Haematopo | 0.110748822 | 8.54057322 | 9.608800179 |
| CVCL_8800 | OCI-Ly3 | CellLine | Lymphoma | Bone Marro | 0.067472638 | 8.520355206 | 9.569145915 |
| CVCL_1889 | SU-DHL-10 | CellLine | Lymphoma | Pleural Effu | 0 | 8.465721742 | 9.087211684 |
| CVCL_1101 | CA46 | CellLine | Lymphoma | Ascites | 0.120540853 | 8.421073835 | 7.947684612 |
| CVCL_1280 | HH [Human | CellLine | Lymphoma | Haematopo | 2.522413872 | 8.396239855 | 9.103942537 |
| CVCL_1064 | A4/Fukuda | CellLine | Lymphoma | Ascites | 0.042867578 | 8.281419919 | 9.971200606 |
| CVCL_1872 | Mino | CellLine | Lymphoma | Haematopo | 0.391359122 | 8.248380709 | 9.368772172 |
| CVCL_1088 | BL-70 | CellLine | Lymphoma | Haematopo | 0.142207055 | 8.200497844 | 9.423298224 |
| CVCL_1325 | Karpas-422 | CellLine | Lymphoma | Pleural Effu | 0.14091868 | 8.153011293 | 8.721040419 |
| CVCL_0017 | U-698-M | CellLine | Lymphoma | upper_aero | 0.01990185 | 8.113667645 | 9.685092631 |
| CVCL_0337 | HuT 78 | CellLine | Lymphoma | Haematopo | 1.599058623 | 7.91905195 | 9.296178842 |
| CVCL_2206 | SU-DHL-6 | CellLine | Lymphoma | Ascites | 0.022556954 | 7.859905357 | 9.066203801 |
| CVCL_0538 | SU-DHL-1 | CellLine | Lymphoma | Pleural Effu | 0 | 7.827145055 | 9.091245742 |
| CVCL_1414 | MJ | CellLine | Lymphoma | Haematopo | 2.346013534 | 7.623514248 | 9.454285653 |
| CVCL_1902 | WSU-DLCL2 | CellLine | Lymphoma | Pleural Effu | 0.043814574 | 7.575563844 | 8.249317818 |
| CVCL_1179 | DoHH2 | CellLine | Lymphoma | Pleural Effu | 0.106086014 | 7.521504702 | 9.121923026 |
| CVCL_0107 | BCP-1 | CellLine | Lymphoma | Haematopo | 0.056983477 | 7.515398775 | 8.826925594 |
| CVCL_1362 | L-540 | CellLine | Lymphoma | Bone Marro | 0.39420279 | 7.507685311 | 8.391585347 |
| CVCL_3611 | Toledo | CellLine | Lymphoma | Haematopo | 0 | 7.394271654 | 9.355284727 |
| CVCL_1087 | BL-41 | CellLine | Lymphoma | Haematopo | 0.126460679 | 7.328722654 | 9.906644044 |
| CVCL_1099 | C8166 | CellLine | Lymphoma | Haematopo | 0.036597628 | 7.214615434 | 9.003615977 |
| CVCL_1885 | Ri-1 | CellLine | Lymphoma | Haematopo | 0.40770922 | 7.208588776 | 9.202424237 |
| CVCL_1290 | HT | CellLine | Lymphoma | Ascites | 0.035646083 | 7.207018329 | 9.152600634 |
| CVCL_1319 | JVM-2 | CellLine | Lymphoma | Haematopo | 0.148827778 | 7.066293709 | 8.536392694 |
| CVCL_3326 | Pfeiffer | CellLine | Lymphoma | Pleural Effu | 0.286423704 | 7.035553352 | 9.021878739 |
| CVCL_2093 | Ki-JK | CellLine | Lymphoma | Pleural Effu | 0.226504133 | 7.006404709 | 9.536762579 |
| CVCL_0009 | HDLM-2 | CellLine | Lymphoma | Pleural Effu | 0.079182072 | 6.826481341 | 7.937857546 |
| CVCL_1735 | SU-DHL-5 | CellLine | Lymphoma | Lymph Noc | 0 | 6.76017046 | 9.328648202 |
| CVCL_0823 | Hs 611.T | CellLine | Lymphoma | Haematopo | 0.135529258 | 6.457182353 | 9.218574017 |
| CVCL_1818 | Granta-519 | CellLine | Lymphoma | Haematopo | 0.425226561 | 6.266075164 | 8.34589012 |
| CVCL_1361 | L-428 | CellLine | Lymphoma | Pleural Effu | 0.361011072 | 6.256959443 | 9.889273066 |
| CVCL_2096 | L-1236 | CellLine | Lymphoma | Haematopo | 0.577341264 | 6.08824719 | 8.834216406 |
| CVCL_3526 | HuT 102 | CellLine | Lymphoma | Lymph Noc | 0.045970288 | 6.084295619 | 8.103733611 |
| CVCL_1170 | DEL | CellLine | Lymphoma | Pleural Effu | 0.201568911 | 5.916383341 | 8.265452716 |
| CVCL_1330 | KM-H2 | CellLine | Lymphoma | Pleural Effu | 0.51959724 | 5.892357136 | 8.897923656 |
| CVCL_1194 | EHEB | CellLine | Lymphoma | Haematopo | 0.128794119 | 5.859144867 | 8.323785196 |
| CVCL_0891 | Hs 751.T | CellLine | Lymphoma | Fibroblast | 0.050252792 | 5.743921608 | 7.852004035 |
| CVCL_1324 | Karpas-299 | CellLine | Lymphoma | Haematopo | 0.267638809 | 5.69276223 | 8.657533252 |
| CVCL_2027 | EB1 | CellLine | Lymphoma | Bone | 0.077495847 | 5.677796879 | 8.537325375 |
| CVCL_0825 | Hs 616.T | CellLine | Lymphoma | Fibroblast | 0.124158315 | 5.214523851 | 7.701326819 |
| CVCL_3806 | TO 175.T | CellLine | Lymphoma | Lymph Noc | 0 | 5.127446175 | 7.582733879 |
| CVCL_2209 | SUP-M2 | CellLine | Lymphoma | Central Ne | 0.166687146 | 4.496340529 | 8.586290879 |
| CVCL_1711 | SR | CellLine | Lymphoma | Pleural Effu | 0.056926097 | 4.453529 | 7.930887397 |

| | | | | | | | |
|---|---|---|---|---|---|---|---|
| CVCL_0018 | D341 Med | CellLine | Medullobla | Central Ne | 0.789143772 | 10.29036491 | 8.15194 |
| CVCL_1155 | D283 Med | CellLine | Medullobla | Abdomen | 0.213496418 | 9.744800679 | 9.02637085 |
| CVCL_1624 | ONS-76 | CellLine | Medullobla | Central Ne | 0 | 5.957288993 | 7.714077542 |
| **CVCL_1167** | **Daoy** | **CellLine** | **Medullobla** | **Central Ne** | **0.523145112** | **4.753715276** | **8.298034275** |
| **CVCL_0019** | **SH-SY5Y** | **CellLine** | **Neuroblast** | **bone_marr** | **4.092247404** | **6.042883253** | **7.824253429** |
| **CVCL_1700** | **SK-N-AS** | **CellLine** | **Neuroblast** | **Bone Marr** | **0.596660656** | **5.410889311** | **8.273219506** |
| **CVCL_0531** | **SK-N-SH** | **CellLine** | **Neuroblast** | **Bone Marr** | **0.797850712** | **4.711308545** | **7.995358064** |
| CVCL_1340 | KP-N-SI9s | CellLine | Neuroblast | Autonomic | 0.686786263 | 2.897990132 | 8.152506334 |
| CVCL_1702 | SK-N-FI | CellLine | Neuroblast | Bone Marr | 5.411570357 | 1.422950813 | 8.469944354 |
| CVCL_IZ34 | HMEL | CellLine | Non-Cance | Breast | 0.273668077 | 8.168624294 | 8.246107081 |
| DZQV | B_NSM | PBMC | Non-Cancerous | | 0 | 7.798721987 | 7.110214347 |
| X9JD4 | Treg | PBMC | Non-Cancerous | | 0 | 7.52751798 | 7.658498279 |
| X925L | Th17 | PBMC | Non-Cancerous | | 0 | 7.47626149 | 7.810450816 |
| X9JD4 | Th17 | PBMC | Non-Cancerous | | 0 | 7.367739119 | 7.747207918 |
| X9JD4 | CD4_naive | PBMC | Non-Cancerous | | 0 | 7.340166399 | 7.785340514 |
| X9JD4 | Th2 | PBMC | Non-Cancerous | | 0 | 7.307339598 | 7.590125388 |
| X9JD4 | CD8_naive | PBMC | Non-Cancerous | | 0 | 7.303387637 | 8.047786963 |
| G4YW | Th17 | PBMC | Non-Cancerous | | 0 | 7.254288946 | 7.65892387 |
| DZQV | MAIT | PBMC | Non-Cancerous | | 0 | 7.230381917 | 7.629411267 |
| DZQV | Treg | PBMC | Non-Cancerous | | 0 | 7.213038489 | 7.607131652 |
| G4YW | CD8_naive | PBMC | Non-Cancerous | | 0 | 7.210046503 | 7.643961575 |
| DZQV | Th17 | PBMC | Non-Cancerous | | 0 | 7.163366915 | 7.450150135 |
| X9JD4 | MAIT | PBMC | Non-Cancerous | | 0 | 7.149752199 | 7.794016535 |
| X9JD4 | TFH | PBMC | Non-Cancerous | | 0 | 7.135487322 | 7.802522763 |
| X925L | CD4_naive | PBMC | Non-Cancerous | | 0 | 7.096180771 | 7.539845944 |
| DZQV | Th1_Th17 | PBMC | Non-Cancerous | | 0 | 7.094093394 | 7.528388548 |
| DZQV | Th2 | PBMC | Non-Cancerous | | 0 | 7.065332191 | 7.636311979 |
| **DZQV** | **Progenitor** | **PBMC** | **Non-Cancerous** | | **3.011471129** | **7.058397353** | **8.189764281** |
| X925L | B_NSM | PBMC | Non-Cancerous | | 0 | 6.996150617 | 6.951870208 |
| X925L | CD8_naive | PBMC | Non-Cancerous | | 0 | 6.899633652 | 7.526767604 |
| DZQV | CD4_naive | PBMC | Non-Cancerous | | 0 | 6.896776074 | 7.658727547 |
| G4YW | CD4_naive | PBMC | Non-Cancerous | | 0 | 6.883533455 | 7.696132538 |
| X925L | Th1_Th17 | PBMC | Non-Cancerous | | 0 | 6.862268873 | 7.381624709 |
| X9JD4 | Th1 | PBMC | Non-Cancerous | | 0 | 6.853488594 | 7.514012065 |
| X925L | Progenitor | PBMC | Non-Cancerous | | 1.142354618 | 6.84729416 | 7.429893179 |
| X9JD4 | Th1_Th17 | PBMC | Non-Cancerous | | 0 | 6.83664561 | 7.620021648 |
| X9JD4 | B_NSM | PBMC | Non-Cancerous | | 0 | 6.770264673 | 7.207695034 |
| X925L | TFH | PBMC | Non-Cancerous | | 0 | 6.732077723 | 7.37171255 |
| X925L | MAIT | PBMC | Non-Cancerous | | 0 | 6.723809851 | 8.015150297 |
| X925L | Treg | PBMC | Non-Cancerous | | 0 | 6.696574796 | 7.743271653 |
| DZQV | CD8_naive | PBMC | Non-Cancerous | | 0 | 6.692433057 | 8.110667097 |
| G4YW | Th1_Th17 | PBMC | Non-Cancerous | | 0 | 6.685562428 | 7.81473799 |
| G4YW | MAIT | PBMC | Non-Cancerous | | 0 | 6.651095668 | 7.632906016 |
| CVCL_WS6 | SALE | CellLine | Non-Cance | Lung | 0.024816786 | 6.626385711 | 7.937541359 |
| G4YW | TFH | PBMC | Non-Cancerous | | 0 | 6.61309511 | 7.446645473 |
| X9JD4 | B_naive | PBMC | Non-Cancerous | | 0 | 6.592023027 | 7.203189702 |
| X9JD4 | CD8_CM | PBMC | Non-Cancerous | | 0 | 6.56267706 | 7.362739448 |
| G4YW | Progenitor | PBMC | Non-Cancerous | | 2.242755837 | 6.540422245 | 7.605436922 |

| | | | | | | |
|---|---|---|---|---|---|---|
| X925L | CD8_CM | PBMC | Non-Cancerous | 0 | 6.524663472 | 7.695428967 |
| G4YW | PBMC | PBMC | Non-Cancerous | 0 | 6.515069361 | 7.404743922 |
| DZQV | Th1 | PBMC | Non-Cancerous | 0 | 6.492019253 | 7.530876039 |
| DZQV | TFH | PBMC | Non-Cancerous | 0 | 6.488367506 | 7.910548117 |
| X925L | Th2 | PBMC | Non-Cancerous | 0 | 6.451520969 | 7.537555816 |
| G4YW | Treg | PBMC | Non-Cancerous | 0 | 6.450631114 | 7.571624179 |
| CVCL_V626 | PrEC LH | CellLine | Non-Cance Prostate | 0 | 6.435435132 | 7.448299342 |
| CVCL_WS7 | NHAHTDD | CellLine | Non-Cance Central Ne | 0 | 6.418983952 | 8.161418014 |
| CVCL_IW56 | OELE | CellLine | Non-Cance Ovary | 0.110326417 | 6.41490847 | 8.347202986 |
| G4YW | Th2 | PBMC | Non-Cancerous | 0 | 6.404405833 | 7.653820013 |
| G4YW | B_NSM | PBMC | Non-Cancerous | 0 | 6.34337442 | 7.137686513 |
| X925L | B_naive | PBMC | Non-Cancerous | 0 | 6.330334163 | 6.834619958 |
| X4DUY | PBMC | PBMC | Non-Cancerous | 0 | 6.297510356 | 7.376921789 |
| X9JD4 | PBMC | PBMC | Non-Cancerous | 0 | 6.280251709 | 7.461512271 |
| X9JD4 | Progenitor | PBMC | Non-Cancerous | 2.644936537 | 6.277768141 | 7.709078159 |
| DZQV | B_naive | PBMC | Non-Cancerous | 0.199625236 | 6.216563282 | 7.060230466 |
| DZQV | PBMC | PBMC | Non-Cancerous | 0 | 6.073512921 | 7.088159622 |
| G4YW | Th1 | PBMC | Non-Cancerous | 0 | 6.066929585 | 7.616460037 |
| G4YW | CD8_CM | PBMC | Non-Cancerous | 0 | 6.020459975 | 7.666336999 |
| DZQV | B_Ex | PBMC | Non-Cancerous | 0 | 5.978546008 | 6.567277366 |
| X925L | Th1 | PBMC | Non-Cancerous | 0 | 5.96443359 | 7.465695038 |
| CVCL_0942 | Hs 840.T | CellLine | Non-Cance Fibroblast | 0.136124968 | 5.923884227 | 8.54358387 |
| DZQV | B_SM | PBMC | Non-Cancerous | 0 | 5.914211914 | 7.132225277 |
| X925L | PBMC | PBMC | Non-Cancerous | 0 | 5.895832724 | 7.288159846 |
| DZQV | CD8_CM | PBMC | Non-Cancerous | 0 | 5.887525539 | 7.967717009 |
| CVCL_WS5 | HEK TE | CellLine | Non-Cance Kidney | 0.146023779 | 5.864628971 | 8.278927535 |
| X925L | B_SM | PBMC | Non-Cancerous | 0 | 5.77591761 | 6.928943497 |
| CVCL_0888 | Hs 742.T | CellLine | Non-Cance Fibroblast | 0.259801016 | 5.762418241 | 7.480375494 |
| X9JD4 | VD2_1 | PBMC | Non-Cancerous | 0 | 5.736461897 | 7.643434854 |
| X9JD4 | B_SM | PBMC | Non-Cancerous | 0 | 5.721150332 | 7.223469082 |
| CVCL_0979 | Hs 888.T | CellLine | Non-Cance Fibroblast | 0.081070963 | 5.673033361 | 8.013788875 |
| G4YW | B_naive | PBMC | Non-Cancerous | 0 | 5.634908319 | 6.994281696 |
| X925L | VD2_1 | PBMC | Non-Cancerous | 0 | 5.595338322 | 7.73098809 |
| CVCL_0882 | Hs 739.T | CellLine | Non-Cance Fibroblast | 0.123660378 | 5.582240563 | 7.440498233 |
| FLWA | PBMC | PBMC | Non-Cancerous | 0 | 5.581735704 | 7.426383856 |
| CVCL_0855 | Hs 698.T | CellLine | Non-Cance Soft Tissue | 0.019055627 | 5.548668034 | 7.893524215 |
| X9JD4 | B_Ex | PBMC | Non-Cancerous | 0 | 5.532322365 | 6.63650978 |
| G4YW | B_SM | PBMC | Non-Cancerous | 0 | 5.527866864 | 7.100519713 |
| CVCL_1031 | Hs 934.T | CellLine | Non-Cance Fibroblast | 0.076951128 | 5.495495862 | 7.953125033 |
| CVCL_0827 | Hs 618.T | CellLine | Non-Cance Fibroblast | 0.099466537 | 5.49408527 | 8.524728575 |
| X925L | B_Ex | PBMC | Non-Cancerous | 0 | 5.464301924 | 6.75296423 |
| CVCL_0819 | Hs 606.T | CellLine | Non-Cance Fibroblast | 0.15365519 | 5.460131821 | 7.5321459 |
| CVCL_0726 | Hs 343.T | CellLine | Non-Cance Fibroblast | 0.159794606 | 5.4557931 | 8.235422846 |
| CVCL_V617 | TIG-3 TD | CellLine | Non-Cance Fibroblast | 0.02554948 | 5.450050841 | 8.10637037 |
| CVCL_1740 | TE 125.T | CellLine | Non-Cance Fibroblast | 0.045844018 | 5.435341737 | 7.464662663 |
| X684C | PBMC | PBMC | Non-Cancerous | 0 | 5.430210982 | 7.289469965 |
| G4YW | B_Ex | PBMC | Non-Cancerous | 0 | 5.377779755 | 6.942242244 |
| G4YW | VD2_1 | PBMC | Non-Cancerous | 0 | 5.357430215 | 7.440321628 |

| | | | | | | |
|---|---|---|---|---|---|---|
| CVCL_1038 | Hs 940.T | CellLine | Non-Cance Fibroblast | 0.117677019 | 5.344545574 | 7.723960498 |
| CVCL_0711 | Hs 274.T | CellLine | Non-Cance Fibroblast | 0.039129831 | 5.327554433 | 7.400946198 |
| CVCL_2255 | HLF-a | CellLine | Non-Cance Fibroblast | 0.122847916 | 5.269260295 | 8.135818246 |
| CVCL_0938 | Hs 834.T | CellLine | Non-Cance Lymph Noc | 0.173771861 | 5.261078092 | 7.849927295 |
| CVCL_3605 | T1-73 | CellLine | Non-Cance Fibroblast | 0.032956556 | 5.198044526 | 7.367570629 |
| CYFZ | PBMC | PBMC | Non-Cancerous | 0 | 5.165159041 | 7.340776948 |
| CVCL_0715 | Hs 281.T | CellLine | Non-Cance Fibroblast | 0.170961067 | 5.15490578 | 7.9295899 |
| CVCL_2182 | RS-5 | CellLine | Non-Cance Fibroblast | 0.609569228 | 5.112086375 | 7.66309396 |
| CVCL_0932 | Hs 821.T | CellLine | Non-Cance Fibroblast | 0.071219452 | 5.11075815 | 7.852434218 |
| CVCL_0698 | Hs 229.T | CellLine | Non-Cance Fibroblast | 0.036848296 | 5.09395564 | 8.470351446 |
| CVCL_0966 | Hs 870.T | CellLine | Non-Cance Fibroblast | 0.073735517 | 5.088149304 | 8.111952257 |
| CVCL_0993 | Hs 895.T | CellLine | Non-Cance Fibroblast | 0.048021479 | 5.085199002 | 7.624431787 |
| CVCL_0846 | Hs 688(A).T | CellLine | Non-Cance Fibroblast | 0.088427731 | 5.083847345 | 7.493686873 |
| CVCL_2019 | DM-3 | CellLine | Non-Cance Fibroblast | 0 | 5.056161682 | 7.925428962 |
| CVCL_0933 | Hs 822.T | CellLine | Non-Cance Fibroblast | 0.14629397 | 5.04790247 | 7.090217111 |
| CVCL_0928 | Hs 819.T | CellLine | Non-Cance Fibroblast | 0.124312046 | 5.009621047 | 7.332307845 |
| FY2H | PBMC | PBMC | Non-Cancerous | 0 | 4.962638684 | 6.923965601 |
| CR3L | PBMC | PBMC | Non-Cancerous | 0 | 4.891211289 | 6.899748787 |
| CVCL_0941 | Hs 839.T | CellLine | Non-Cance Fibroblast | 0.059076557 | 4.876445558 | 7.885111745 |
| G4YW | CD8_EM | PBMC | Non-Cancerous | 0 | 4.853277139 | 7.905990253 |
| CZJE | PBMC | PBMC | Non-Cancerous | 0 | 4.835819715 | 7.189747738 |
| X9JD4 | CD4_TE | PBMC | Non-Cancerous | 0 | 4.827608498 | 7.576342576 |
| CVCL_1743 | TE 159.T | CellLine | Non-Cance Fibroblast | 0.020890285 | 4.762469866 | 7.313563975 |
| CVCL_0863 | Hs 706.T | CellLine | Non-Cance Fibroblast | 0.063238803 | 4.748366742 | 7.850765988 |
| DZQV | I_mono | PBMC | Non-Cancerous | 0 | 4.733633652 | 6.247854238 |
| X9JD4 | CD8_EM | PBMC | Non-Cancerous | 0 | 4.713820671 | 7.762586676 |
| CVCL_0878 | Hs 737.T | CellLine | Non-Cance Fibroblast | 0.146348344 | 4.693486486 | 7.394946705 |
| G4YW | C_mono | PBMC | Non-Cancerous | 0 | 4.597162705 | 6.738423332 |
| CVCL_0814 | Hs 600.T | CellLine | Non-Cance Fibroblast | 0.07046562 | 4.584556541 | 7.734318739 |
| X453W | PBMC | PBMC | Non-Cancerous | 0 | 4.578248072 | 7.565554528 |
| X925L | VD2_2 | PBMC | Non-Cancerous | 0 | 4.545987217 | 7.531696791 |
| DZQV | Plasmablas | PBMC | Non-Cancerous | 0 | 4.528343458 | 6.523666203 |
| X9JD4 | C_mono | PBMC | Non-Cancerous | 0 | 4.501930761 | 6.986196973 |
| DZQV | CD8_EM | PBMC | Non-Cancerous | 0 | 4.424400047 | 7.806867151 |
| CVCL_0959 | Hs 863.T | CellLine | Non-Cance Fibroblast | 0.079512226 | 4.422912439 | 7.945634571 |
| CVCL_0707 | Hs 255.T | CellLine | Non-Cance Fibroblast | 0.228300412 | 4.420061694 | 6.869996803 |
| X925L | CD8_EM | PBMC | Non-Cancerous | 0 | 4.386061472 | 8.103751285 |
| CVCL_0836 | Hs 675.T | CellLine | Non-Cance Fibroblast | 0.193474902 | 4.290227018 | 7.852063164 |
| X925L | I_mono | PBMC | Non-Cancerous | 0 | 4.266521591 | 6.459209131 |
| X36TS | PBMC | PBMC | Non-Cancerous | 0.079461948 | 4.24410448 | 7.025690495 |
| X9JD4 | VD2_2 | PBMC | Non-Cancerous | 0 | 4.240901059 | 7.937400481 |
| G4YW | Plasmablas | PBMC | Non-Cancerous | 0 | 4.22663661 | 6.074729106 |
| DZQV | NC_mono | PBMC | Non-Cancerous | 0 | 4.182891069 | 6.432336276 |
| X9JD4 | Plasmablas | PBMC | Non-Cancerous | 0 | 4.170441145 | 5.821109928 |
| X925L | CD4_TE | PBMC | Non-Cancerous | 0 | 4.117450897 | 7.13492825 |
| X925L | Plasmablas | PBMC | Non-Cancerous | 0 | 4.013883859 | 6.066341532 |
| X9JD4 | mDC | PBMC | Non-Cancerous | 0 | 4.009700145 | 7.473008705 |
| X9JD4 | I_mono | PBMC | Non-Cancerous | 0 | 3.982179641 | 7.033991157 |

| | | | | | | | |
|---|---|---|---|---|---|---|---|
| X925L | NC_mono | PBMC | Non-Cancerous | | 0 | 3.944667356 | 6.680501391 |
| G4YW | NC_mono | PBMC | Non-Cancerous | | 0 | 3.938023785 | 7.151662194 |
| G4YW | I_mono | PBMC | Non-Cancerous | | 0 | 3.800325779 | 6.916879396 |
| G4YW | mDC | PBMC | Non-Cancerous | | 0 | 3.681645826 | 7.503999248 |
| X925L | CD8_TE | PBMC | Non-Cancerous | | 0 | 3.50150091 | 7.709965362 |
| DZQV | VD2_1 | PBMC | Non-Cancerous | | 0 | 3.493629971 | 7.674072186 |
| DZQV | C_mono | PBMC | Non-Cancerous | | 0 | 3.478021772 | 6.585232981 |
| X9JD4 | NC_mono | PBMC | Non-Cancerous | | 0 | 3.465503381 | 7.022593189 |
| X925L | C_mono | PBMC | Non-Cancerous | | 0 | 3.376486181 | 6.839859215 |
| X9JD4 | CD8_TE | PBMC | Non-Cancerous | | 0 | 3.366383648 | 7.792269204 |
| DZQV | mDC | PBMC | Non-Cancerous | | 0 | 3.353967225 | 7.414460365 |
| G4YW | VD2_2 | PBMC | Non-Cancerous | | 0 | 3.293296585 | 7.5664966 |
| X9JD4 | NK | PBMC | Non-Cancerous | | 0 | 2.789896684 | 8.020561872 |
| DZQV | VD2_2 | PBMC | Non-Cancerous | | 0 | 2.758522061 | 7.765929975 |
| G4YW | CD8_TE | PBMC | Non-Cancerous | | 0 | 2.728790944 | 7.948438664 |
| CVCL_0660 | Hs 172.T | CellLine | Non-Cance | Fibroblast | 2.937412299 | 2.718170941 | 7.076923893 |
| X925L | mDC | PBMC | Non-Cancerous | | 0 | 2.42214956 | 6.903245956 |
| DZQV | CD8_TE | PBMC | Non-Cancerous | | 0 | 2.257390438 | 7.667968954 |
| DZQV | NK | PBMC | Non-Cancerous | | 0 | 1.912858349 | 7.988476447 |
| X925L | NK | PBMC | Non-Cancerous | | 0 | 1.735059834 | 7.587290574 |
| G4YW | NK | PBMC | Non-Cancerous | | 0 | 1.705473652 | 7.964946586 |
| DZQV | pDC | PBMC | Non-Cancerous | | 0 | 0.594428918 | 7.752226216 |
| X925L | Neutrophils | PBMC | Non-Cancerous | | 0 | 0.465073837 | 2.162993056 |
| X9JD4 | Basophils | PBMC | Non-Cancerous | | 0 | 0.443821979 | 6.473639518 |
| DZQV | Neutrophils | PBMC | Non-Cancerous | | 0 | 0.411463126 | 2.234210361 |
| X925L | Basophils | PBMC | Non-Cancerous | | 0 | 0.345373665 | 6.974286858 |
| G4YW | pDC | PBMC | Non-Cancerous | | 0.344456984 | 0.269629257 | 7.395163764 |
| G4YW | Basophils | PBMC | Non-Cancerous | | 0 | 0.268277479 | 6.160302668 |
| G4YW | Neutrophils | PBMC | Non-Cancerous | | 0 | 0.200583448 | 2.590442496 |
| X9JD4 | pDC | PBMC | Non-Cancerous | | 0 | 0.131012159 | 7.686598571 |
| X9JD4 | Neutrophils | PBMC | Non-Cancerous | | 0 | 0.094022927 | 2.99305531 |
| X925L | pDC | PBMC | Non-Cancerous | | 0.601547248 | 0 | 7.568787543 |
| DZQV | Basophils | PBMC | Non-Cancerous | | 0 | 0 | 5.958944959 |
